# A citizen-science-enabled catalogue of the vaginal microbiome and associated factors

**DOI:** 10.1038/s41564-023-01500-0

**Published:** 2023-10-26

**Authors:** Sarah Lebeer, Sarah Ahannach, Thies Gehrmann, Stijn Wittouck, Tom Eilers, Eline Oerlemans, Sandra Condori, Jelle Dillen, Irina Spacova, Leonore Vander Donck, Caroline Masquillier, Camille Nina Allonsius, Peter A. Bron, Wannes Van Beeck, Charlotte De Backer, Gilbert Donders, Veronique Verhoeven

**Affiliations:** 1 Department of Bioscience Engineering, Research Group Environmental Ecology and Applied Microbiology, University of Antwerp, Antwerp, Belgium; 2 Department of Sociology, Center for Population, Family and Health, University of Antwerp, Antwerp, Belgium; 3 Department Communication Sciences, University of Antwerp, Antwerp, Belgium; 4 Department of Obstetrics and Gynaecology, University Hospital Antwerp, Edegem, Belgium; 5 Regional Hospital Heilig Hart, Tienen, Belgium; 6 Femicare Clinical Research for Women, Tienen, Belgium; 7 Department of Family Medicine and Population Health, University of Antwerp, Antwerp, Belgium

**Keywords:** Microbiology, Molecular biology

## Abstract

Understanding the composition and function of the vaginal microbiome is crucial for reproductive and overall health. Here we established the Isala citizen-science project to analyse the vaginal microbiomes of 3,345 women in Belgium (18–98 years) through self-sampling, 16S amplicon sequencing and extensive questionnaires. The overall vaginal microbiome composition was strongly tied to age, childbirth and menstrual cycle phase. *Lactobacillus* species dominated 78% of the vaginal samples. Specific bacterial taxa also showed to co-occur in modules based on network correlation analysis. Notably, the module containing *Lactobacillus crispatus*, *Lactobacillus jensenii* and *Limosilactobacillus* taxa was positively linked to oestrogen levels and contraceptive use and negatively linked to childbirth and breastfeeding. Other modules, named after abundant taxa (*Gardnerella*, *Prevotella* and *Bacteroides*), correlated with multiple partners, menopause, menstrual hygiene and contraceptive use. With this resource-rich vaginal microbiome map and associated health, life-course, lifestyle and dietary factors, we provide unique data and insights for follow-up clinical and mechanistic research.

## Abstract (web summary)

A citizen-science approach is used to map the vaginal microbiome of 3,345 women and identify associations with health, life-course and lifestyle factors.

## Main

For more than a century, a healthy vagina has been considered a simple ecosystem dominated by rod-shaped, Gram-positive bacteria, nowadays known as lactobacilli^1^. The dominance of lactobacilli in the vagina is mainly observed during reproductive age, where *Lactobacillus* bacteria play a pivotal role in maintaining a healthy vaginal ecosystem. These bacteria produce lactic acid as major antimicrobial molecule: this mild acid can inhibit unwanted, often sexually transmittable, pathogens that pose a health risk to a woman, her partner and her baby during pregnancy and delivery^2^. More recently, specific *Lactobacillus* species have been identified to also produce various other molecules pivotal for a balanced interaction with the vaginal cells and promotion of optimal functioning of this organ^3^. For more than a decade, insights in the composition and functionality of the vaginal microbiome are generated via next-generation sequencing^4^. These studies typically describe five community state types (CSTs), of which four are dominated by one specific *Lactobacillus* species^4^. *L.* *crispatus*, *L.* *gasseri*, *L.* *iners* and *L.* *jensenii* are dominant in CST I, II, III and V, respectively. These four species all belong to the updated *Lactobacillus* genus strictu sensu and are evolutionarily distinct from lactobacilli commonly used as gut probiotics or present in fermented food^5^. In CST IV, a mixture of anaerobes is generally observed, which typically include *Gardnerella* (recently emended^6^), *Atopobium* (recently reclassified as *Fannyhessea vaginae*^7^), *Prevotella* (recently updated^8^) and *Finegoldia*^9^. This CST IV is associated with conditions such as bacterial vaginosis (BV), but is also frequently observed in women without symptoms. The CST framework is continuously updated, for example in the VAginaL community state typE Nearest CentroId clAssifier (VALENCIA) study^10^.

Humans appear to be the only animals with a vagina mostly dominated by *Lactobacillus* taxa starting from puberty^11^. It is not well understood why this is the case, but it is associated with the typical human menstrual cycle. Oestrogen particularly seems to play a key role in promoting the accumulation of glycogen by vaginal epithelial cells as carbohydrate source for fermentation by lactobacilli. Moreover, the typical human agricultural diet and the strong antimicrobial capacity of lactobacilli, which can protect the limited offspring of humans from infections, have been suggested to play a role^12^. Studies in North America^13^, Scandinavia^14^ and sub-Saharan African countries such as South Africa^15^ and Kenya^16^ have observed differences in the vaginal microbiome composition across the different populations, but the study cohorts were small to medium scale and the relative importance of lifestyle was generally not well considered.

In this Resource, we report on our citizen-science-based self-sampling study, titled ‘Isala’, honouring Isala Van Diest as the first female doctor in Belgium. In a co-creative way, volunteers provided samples and data, proposed research objectives and survey questions, and helped to disseminate and interpret the objectives and results through different media platforms. We aimed to jointly break taboos on women’s and vaginal health and provide insights in how the occurrence of lactobacilli and other vaginal bacteria is associated with lifestyle and life-course events.

## Results

### Dominance of *Lactobacillus* taxa

**Fig. 1:**
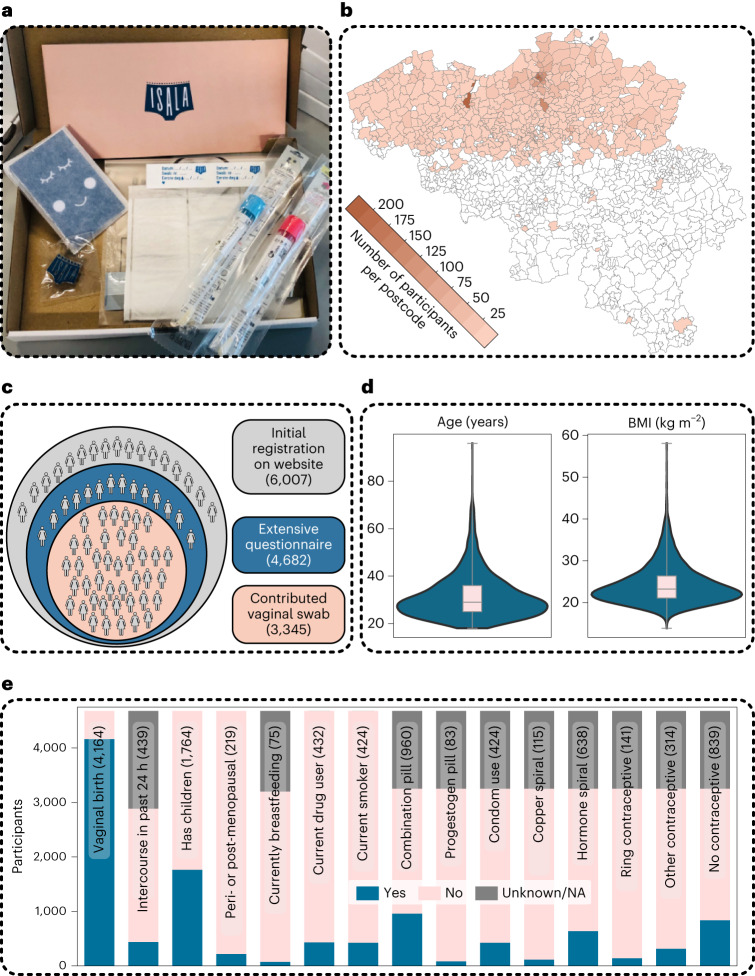
Characterization of the Isala study cohort and key physiological, behavioural, lifestyle and environmental factors of the participating women. **a**, The self-sampling kit sent to the participants via the national postal service. **b**, Geographical overview of the participants who sent in samples for this project, by overlaying their zip codes on a map of all Belgian municipalities. Darker colours represent higher numbers of participants with that specific zip code. As only participants who understood Dutch were allowed to participate, most participants were from the Northern part of Belgium. **c**, An overview of the population cohort that registered within 10 days after the first announcement, with their different citizen-science roles in the Isala project: minimal involvement by expressing online interest as potential donor via website and answering five questions on age, pregnancy, contraceptive use, country of living (for the past 3 years) and zip code (grey), partial involvement by filling out the extensive survey (blue) and full involvement as donors with 24-h follow-up survey (pink). **d**,**e**, The distribution of a selection of the survey variables: age and BMI (median and interquartile range (IQR) represented by the boxplot within the violin plot where the whiskers represent the distribution minima and maxima, *n* = 4,682) (**d**), reported contraceptive use of the whole cohort and a subset of the binary variables (**e**). The grey segments indicate participants who either did not know the answer to the question or did not respond to the second survey. The exact number of participants who answered ‘yes’ is included between brackets. NA, not applicable.

We initially aimed for 200 participants, but the enthusiasm for participation was overwhelming and 6,007 participants registered on the website within 10 days, after which the call was closed. All registrants were invited to fill in an extensive survey (Fig. 1a–c). This survey was adapted from validated quality-of-life surveys, the Mental Health Inventory-5 (ref. ^17^) and a Food Frequency Questionnaire^18^ and complemented with questions on hygiene practices, sexual lifestyle and other life-course events inspired by citizens and non-profit organizations participating in our advisory board (Supplementary Text 1 and Fig. 1d,e). A total of 4,682 participants completed the questionnaires (average time to completion of 49 min), of which 3,345 delivered vaginal samples (Fig. 1a) between July and October 2020.

**Fig. 2:**
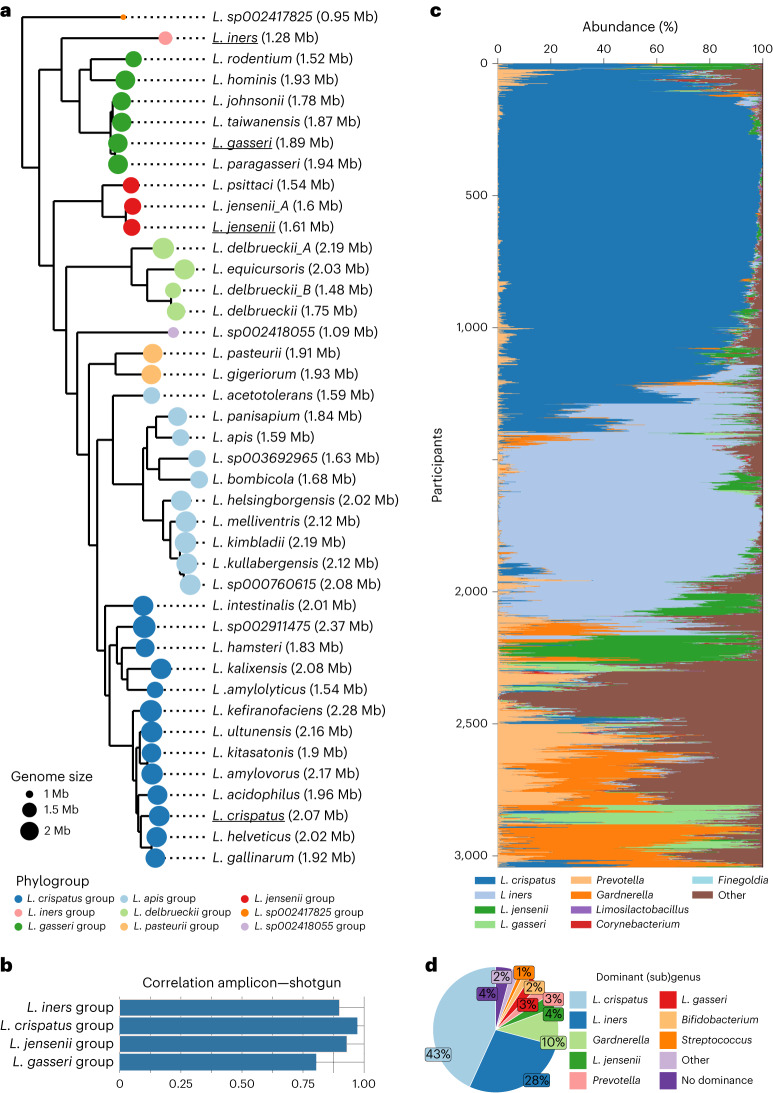
Overview of the most abundant taxa in the vaginal microbiome of the Isala cohort with focus on the *Lactobacillus* taxa. **a**, Maximum-likelihood phylogeny of species of the genus *Lactobacillus* inferred from the amino acid sequences of 100 single-copy core genes. Colours indicate the nine defined subgenera used in this study. Bold tip labels indicate representative species of the subgenera. Species names were taken from the Genome Taxonomy Database^48^, which splits species that are very diverse, yielding, for example*, L. delbrueckii*_A and *L. jensenii*_A, the latter recently identified as *L. mulieris*^69^. The size of the circles reflects the genome size (with the average genome size also shown between brackets). The underlined species names are the species names of the four typical vaginal *Lactobacillus* species, after which the subgenera are named. **b**, Validation of the 16S amplicon sequencing pipeline, including classification to *Lactobacillus* subgenera, with shotgun sequencing data (*n* = 18, Supplementary Fig. 2). For the typical four *Lactobacillus* subgenera, the Spearman correlations between their relative abundances in the amplicon and shotgun samples are shown. **c**, Stacked bar chart describing the microbiome composition of the Isala participants in terms of the nine taxa being first or second most abundant in at least 100 samples. **d**, Occurrence of the most dominant taxa based on the highest taxonomic resolution possible. Dominance was defined as the most abundant taxon that constituted at least 30% of the profile. ‘Other’ refers to the number of samples with a different (sub)genus dominant; ‘No dominance’ refers to the number of samples where no single (sub)genus reached at least 30% relative abundance.

DNA derived from 3,196 samples (95.5%) passed quality control, resulting in 82 million high-quality V4 16S ribosomal RNA (rRNA) read pairs. Subsequently, these V4 amplicon reads were classified up to subgenus level. Hereto, the *Lactobacillus* genus was divided into subgenera based on a high-quality core genome phylogeny (Fig. 2a and Supplementary Fig. 1). This resulted in nine subgenera, four of which included the four main vaginal *Lactobacillus* species (Fig. 2b). This *Lactobacillus* subgenus-level classification of V4 amplicon sequences was validated by shotgun metagenomic sequencing of a subset of samples (*n* = 18) and by classifying V4 reads extracted from 1,121 publicly available whole genomes. For the four subgenera most relevant in the vaginal environment, relative abundances correlated strongly between sequencing techniques (Fig. 2b and Supplementary Fig. 2). V4 sequences from whole genomes were all correctly classified, except sequences from the *Lactobacillus* *delbrueckii* group, but they are not commonly found in the vagina based on the VIRGO metagenomic data^19^ (Supplementary Fig. 3).

Subsequently, for each sample, the dominant (sub)genus was determined as the (sub)genus with the largest relative abundance over 30%. The *L. crispatus* group dominated the largest number of samples (43.2%), followed by the *L. iners* group (27.7%) and *Gardnerella* (9.8%) (Fig. 2c). The *L. jensenii* group (3.5%), *Prevotella* (421 amplicon sequence variants (ASVs)) (3.4%), the *L. gasseri* group (3.2%), *Bifidobacterium* (1.8%) and *Streptococcus* (1.2%) were dominant in much smaller subsets (Fig. 2d). Participants received this information about their dominant vaginal bacterium, as well as the percentages of the eight major taxa as personal microbiome profiles, complemented with fine-tuned texts geared towards non-microbiology experts (Supplementary Figs. 4 and 5).

### Vaginal community structure

**Fig. 3:**
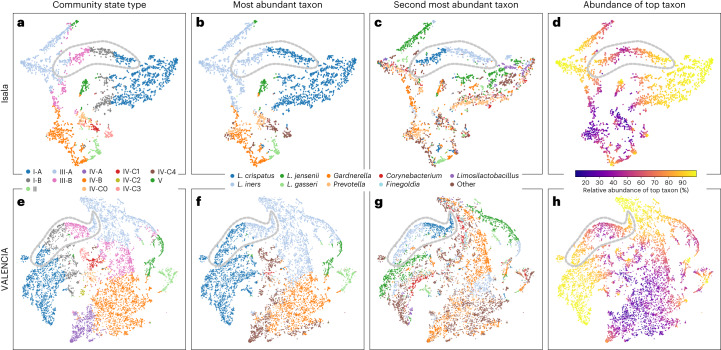
Vaginal microbiome structure of the Isala and VALENCIA cohorts. **a**–**d**, *t*-SNE plot of microbiome samples in the Isala study; samples are coloured by the 13 CSTs as defined by VALENCIA (**a**)^10^. CST I—*L. crispatus*-dominated (A, high relative abundance; B, lower relative abundance), CST II—*L. gasseri*-dominated, CST III—*L. iners*-dominated (A, high relative abundance; B, lower relative abundance) and CST V—*L. jensenii*-dominated. CST IV-A—Candidatus *Lachnocurva vaginae* (BVAB1, identified up to the genus level as EU728721_g in the Isala data) with low abundance of *G.* *vaginalis*. CST IV-B—*G. vaginalis* with low relative abundance of Ca. *L. vaginae*. CST IV-C0—*Prevotella*, CST IV-C1—*Streptococcus*, CST IV-C2—*Enterococcus*-dominated, CST IV-C3—*Bifidobacterium*-dominated and CST IV-C4—*Staphylococcus*-dominated. Samples are coloured by the most abundant (sub)genus (**b**). Samples are coloured by the second most abundant (sub)genus (**c**). Samples are coloured by the largest relative abundance level in each sample (**d**). **e**–**h**, *t*-SNE plot of microbiome samples of the VALENCIA dataset (multi-temporal samples per participant included), coloured by the CSTs (**e**). Samples of the VALENCIA dataset coloured by the most dominant genus (**f**), by the second most dominant genus (**g**) and by the largest relative abundance level in each sample (**h**). In all plots, the grey dashed outlines indicate the ‘bridge’ regions samples dominated by *L. iners* and *L. crispatus*.

To identify patterns in the microbiome compositions of the vaginal microbiota in our cohort, samples were embedded in a two-dimensional space using *t*-distributed stochastic neighbour embedding (*t*-SNE)^20^ (Fig. 3), uniform manifold approximation and projection (Supplementary Fig. 6) and principal coordinates analysis (Supplementary Fig. 7). *t*-SNE is a dimensionality reduction method that prioritizes the preservation of local structure rather than global structure. Several regions were observed in the *t*-SNE plot that broadly corresponded to the 13 previously described CSTs^10^ (Fig. 3a and Supplementary Table 2). These CSTs were connected by intermediate or bridging regions, such as the *L. crispatus* and *L. iners* high-density regions connected by samples with *L. crispatus* and *L. iners* as the two most abundant taxa (Fig. 3b,c and Supplementary Fig. 8). This supports the notion that the previously described CSTs are not distinct possibilities in vaginal community composition but rather form a continuum (Supplementary Fig. 9). This became especially apparent when visualizing the samples based on the second most dominant (sub)genus (Fig. 3c) and the relative abundance of the top (sub)genus (Fig. 3d). A proportion of 335/3,196 (10.4%) samples had at least two taxa with a relative abundance of at least 30% each. Analysis of the VALENCIA^10^ and Vaginal Human Microbiome Project (VaHMP) datasets^13^ (Fig. 3e–h and Supplementary Fig. 10) also pointed at frequent intermediate regions. The distributions of beta diversities within and between CSTs in our dataset and the VALENCIA dataset also showed to be multimodal (Supplementary Figs. 8 and 9). This indicates further structure within the previously defined CSTs^10^.

### Modules of correlated bacterial taxa in the vagina

**Fig. 4:**
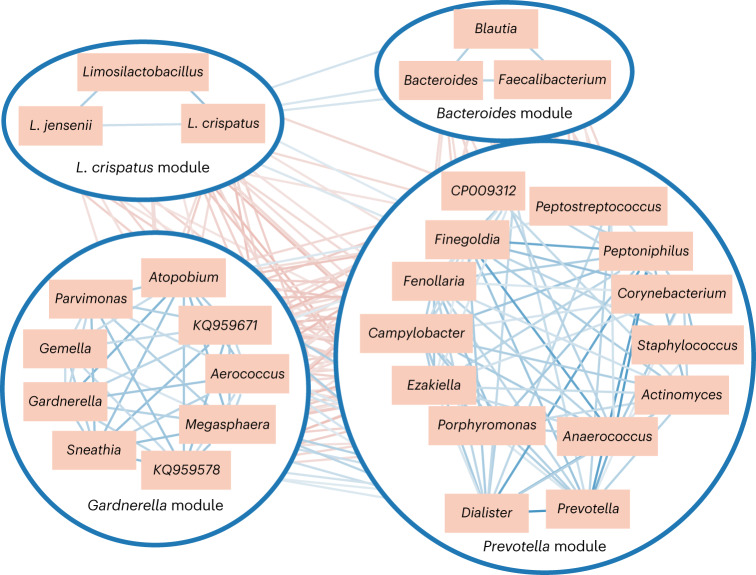
Four main modules of interacting vaginal bacterial taxa as defined by a compositional correlation analysis and consisting of a minimum of three (sub)genera with a biologically plausible relationship. Modules are enclosed in blue circles. Positive and negative correlations are represented in blue and red, respectively. Thickness of the line indicates the strength of the correlation. Quantitative correlations are given in Supplementary Fig. 11. Validation was done with the VALENCIA and VaHMP datasets. Taxa that are not part of modules, such as *L. iners and L. gasseri*, are not shown.

We next investigated how the different bacterial (sub)genera in the vagina were correlated in their abundance across samples. We performed compositional correlation network analyses and discovered modules of co-abundant vaginal taxa (via Sparse Correlations for Compositional data (SparCC)^21^ and Markov clustering (MCL)^22^; Methods and Supplementary Table 3). Four guilds or modules of correlated taxa containing at least three different (sub)genera were distinguished and named after the most abundant (sub)genus per module (Fig. 4). The constituent taxa of the *L. crispatus* module showed negative correlations (*r* between −0.27 and −0.11, and between −0.10 and −0.27, respectively) with the taxa in the *Gardnerella* and *Prevotella* modules. *Prevotella* and *Dialister* (from the *Prevotella* module) showed positive correlations with the *Gardnerella* module (*r* = 0.10–0.32), while the other taxa of these modules showed negative correlations. The *Bacteroides* module was positively correlated with the *L. crispatus* module (*r* = 0.13–0.15). We hypothesize that this latter gut taxa-containing module reflects the biological existence of a gut–vagina axis due to the proximity of the anus and vulva. The *L. iners* group showed a relationship only with the genus *Ureaplasma* (*r* = 0.20). This was in contrast to what we expected on the basis of its small genome size that could suggest it needs metabolic cooperation with other vaginal bacteria to compensate for its lack of specific relevant functions (Fig. 2a). Notably, *Bifidobacterium*, *Streptococcus* and the *L. gasseri* group also did not show strong correlations with other taxa, so they did not appear to occur in a module with at least three different taxa. To validate these modules and bacterial relationships in the vagina, we also calculated SparCC correlations for the VALENCIA^10^ and VaHMP datasets (Supplementary Fig. 11) and found a striking concordance (*r* = 0.39 and 0.47, respectively, *P* < 0.05, Mantel test). When only considering the taxa present in the four modules, the concordance was even stronger (*r* = 0.61 and 0.67, respectively, *P* < 0.05).

In the *L. crispatus* module, a moderate correlation between *Limosilactobacillus* and the *L. crispatus* (*r* = 0.40) and *L. jensenii* groups (*r* = 0.32) was observed. This prompted us to have a closer look at *Limosilactobacillus* taxa. In our large dataset, *Limosilactobacillus* taxa did not show a high average relative abundance (0.4%), but had a surprisingly high prevalence of 47.6% (Supplementary Table 1). Eight *Limosilactobacillus* ASVs had a prevalence of at least 1% (Supplementary Fig. 12). Six of them could be assigned to a *Limosilactobacillus reuteri* group (prevalence of 38.1% or 1,218/3,196), which includes *L. reuteri*, *Limosilactobacillus vaginalis* and nine other species. In addition, one ASV showed a clear match with *Limosilactobacillus coleohominis* (prevalence of 10.1% or 322/3,196) and another with *Limosilactobacillus fermentum* (prevalence of 1.5% or 48/3,196). Inspection of the vaginal metagenomes of the VIRGO metastudy^19^ confirmed *L. coleohominis* (25%), *L. vaginalis* (20%) and *L. fermentum* (1%) to be prevalent species in the vagina.

### Associations of health, life course and lifestyle with the vaginal microbiome

**Fig. 5:**
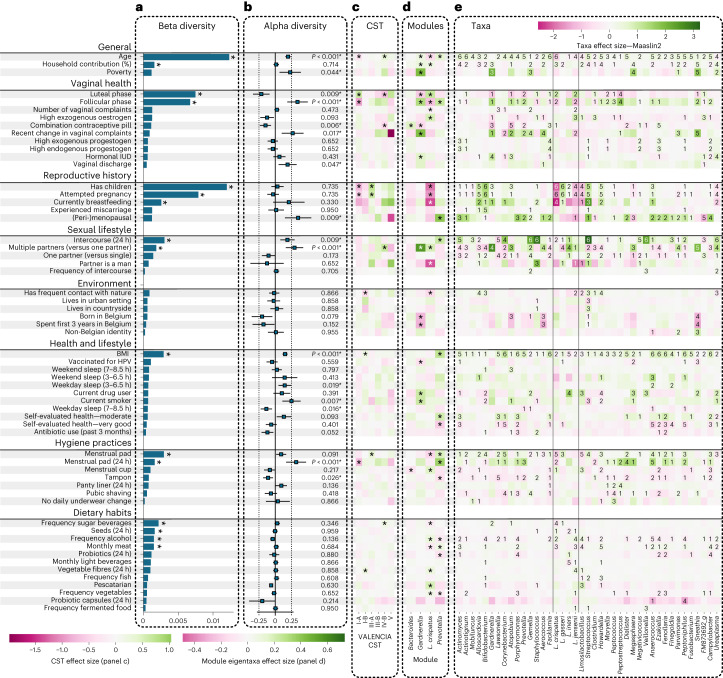
Statistical analysis of the association of different personal, reproductive, lifestyle, health, environmental, hygiene and dietary factors with the vaginal microbiome space. **a**–**e**, Each panel displays associations on different levels of the microbiome: the effect on beta diversity between the samples (Adonis test) (**a**), the effect on the alpha diversity (Shannon index) of the samples (linear model; centre is the effect size, error bars represent 95% confidence inteval, *P* values are indicated on the right) (**b**), the effect on the abundances on six VALENCIA CSTs, which have at least 120 samples in each group (CST I-A, I-B, III-A, III-B, IV-B and V) (**c**), the effect on the module eigentaxa (**d**), and associations of specific taxa (**e**). Asterisks represent significant associations (*q* < 0.05; colour difference between white and black asterisks is for visualization purposes). For the individual taxa tested, the number refers to the number of pipelines that indicate a significant association with the self-reported data: ALDeX2, ANCOM-BC, DESeq, limma, Maaslin2 and a linear regression on the CLR-transformed abundance data. The number of samples for each question was almost the entire study (*n* = 3,043 participants). Due to missing data or specific comparisons, this can deviate slightly: detailed counts and *P* values are provided in Supplementary Table 2. All tests were adjusted for multiple comparisons with Benjamini–Hochberg procedure.

We then analysed the association of self-reported data (Supplementary Text 1 and Supplementary Table 4) with key features of the vaginal microbiome at five levels of abstraction: (1) beta diversity, (2) alpha diversity (Shannon), (3) the VALENCIA CSTs with at least 120 samples in our cohort, (4) eigentaxa for our four modules of at least three intercorrelated taxa (Methods and Supplementary Fig. 13) and (5) 45 (sub)genera selected on the basis of presence in at least 10% of all participants, analysed by six distinct pipelines^23^, with the consensus reported (in at least three tools) (Fig. 5 and Supplementary Table 4 for specific values). Each test at every level was adjusted for technical confounders, age, recent sexual intercourse and antibiotic use in the past 3 months. Importantly, no significant impact was identified for transport time and sampling season on the first six principal components of the abundance data, nor on the beta diversity (permutational multivariate analysis of variance test, *P* > 0.05, Supplementary Tables 5 and 6 and Supplementary Fig. 14). The *L. crispatus* module was clearly positively associated (effect size 0.24, *P* < 0.05) with the luteal phase of the menstrual cycle (Methods and Supplementary Fig. 15) and estimated high exogenous oestrogen use as contraceptive method (combination pill, vaginal ring or patch). The *Gardnerella* module was positively associated with the follicular phase of the menstrual cycle, recent change in vaginal complaints and a hormonal intra-uterine device (IUD) containing only progestin, and was negatively associated with the oral combination contraceptive pill. The *Prevotella* module was associated with menopause and the follicular phase of the menstrual cycle. The CSTs tested here were not associated with most of the host covariates tested (17 factors found with a significance of *P* < 0.05 for CSTs compared with 50 factors for eigentaxa of the modules; Fig. 5). Intermediate regions between CSTs, such as the bridge indicated in Fig. 3a between CST I and III, did show some additional associations, including associations with drug use and coffee drinking (Supplementary Fig. 8). This further indicates that the current CST partitioning is not the most powerful way to investigate associations between lifestyle and the composition of the vaginal microbiome, and we decided to focus on the other parameters in the remainder of the analyses.

Besides age (*R*^2^ = 0.0123), having had children showed the strongest association with beta diversity. It was significantly negatively associated with the abundance levels of the *L. crispatus* module and its three taxa, and positively with the abundance levels of the *L. gasseri* group, *Bifidobacterium* (*breve* and *longum*), *Alloscardovia* and *Streptococcus* (five to six tools). Intercourse in the past 24 h was significantly associated with beta diversity (*R*^2^ = 0.003), a higher alpha diversity and higher levels of the *Prevotella* module and *Staphylococcus*, *Streptococcus*, *Gemella* and *Veillonella*, among others. We also investigated the associations of partnership with the vaginal microbiome. Compared with being sexually inactive, having one partner was not correlated with the alpha and beta diversity, but with some taxa, such as *Gemella*. Having multiple partners was significantly linked to beta diversity, higher alpha diversity and higher levels of the *Gardnerella* module when compared with having one partner, but also higher levels of the *L. crispatus* module, as well as higher levels of *Gardnerella*, *Atopobium*, *Aerococcus*, *L. iners*, *Sneathia* and *Ureaplasma*. Having a male compared with a female partner was associated with lower levels of the *L. crispatus* module, but only showed associations with *Staphylococcus* at the level of individual genera. Of note, having been vaccinated against human papillomavirus (HPV) was associated with lower levels of the *Gardnerella* module, but no associations with specific taxa.

Per request of our participants, we also studied associations for menstrual hygienic products and hygienic habits. The menstrual cup (used by 716 of the 3,043 participants) was positively associated with the *L. crispatus* module, while menstrual pads were negatively associated with the *L. crispatus* module and an increase in the *Prevotella* module, especially when used in the past 48 h. Using a tampon was associated with a decrease of the *Prevotella* module and less *Lawsonella* (CP00931). Pubic shaving showed associations with individual taxa such as lower levels of *Corynebacterium*, *Porphyromonas* and *Peptococcus* and enhanced *Gemella*. Changing underwear less than daily was associated with increased levels of *Peptococcus*. Finally, we studied associations between body mass index (BMI), dietary habits and the vaginal microbiome. BMI was significantly associated with beta diversity (*R*^2^ = 0.0029, *P* < 0.05), higher alpha diversity, higher levels of bacteria in the *Prevotella* module, less *L. iners* and an increase in many other individual taxa. The consumption of sugary beverages was associated with beta diversity with limited effect size (*R* = 0.00055) and with lower levels of the *L. crispatus* module and of *L. crispatus* individually (based on four tools). A high portion of seed consumption (rich in phytoestrogens) in the past 24 h was significantly associated with beta diversity, but not with specific taxa or modules. High frequency of vegetable consumption and its associated fibres, particularly in the past 24 h, and being pescatarian were associated with an increase of the *L. crispatus* module. Ethanol consumption in the past 24 h was associated with higher levels of *L. crispatus* and the *Prevotella* module. Meat consumption was linked to lower levels of the *L. crispatus* module and higher levels of the *Prevotella* module. Significantly lower levels of the *Prevotella* module taxa occurred when probiotic capsules were consumed in the past 24 h.

The combination of all significant factors studied here explained 10.4% of the variation in the vaginal microbiome. This is a considerable percentage compared with other microbiome studies (for instance, 7.63% of the variations could be explained by covariates in a similar study on the gut microbiome in the Belgian population^24^). However, it also shows that a lot is still unknown. Consequently, it requires careful communication to participants to avoid overstating the biological consequences of these individual associations (Supplementary Fig. 16).

## Discussion

This Isala citizen-science study involved intimate self-sampling in the privacy of a home setting. This undoubtedly had a positive impact on the number of women willing to participate, resulting in a large diverse set of samples with sufficient variation to study key health and lifestyle parameters. In addition, the enthusiasm of the participating women to provide self-taken samples for scientific purposes should encourage other researchers, health professionals, policy makers and other stakeholders to further explore the potential of self-sampling for key diagnostic purposes, such as for HPV and sexually transmitted infection testing^25^. Nevertheless, we acknowledge limitations, including that our remote self-sampling strategy disallowed collection of blood samples, clinical examinations and collection of host genetics data. In addition, a citizen-science approach is not a population study. Self-selection bias and a cohort that represents only a subset of society were anticipated limitations. These were outweighed by the major strength of our study, namely that the citizen-science approach allowed us to recruit women outside clinical settings despite the taboo topic and request for intimate samples and data. This ‘positive selection towards generally healthy women’ is well reflected in the health status of our participants: the majority self-reported good health using the broad World Health Organization definition, including no vaginal symptoms (69%).

Of the 3,345 women who donated swabs, 78% were dominated by *Lactobacillus* taxa, with *L. crispatus* (43.2%) and *L. iners* (27.7%) as most dominant species. This *L. iners* prevalence in complaint-free women suggests that this species is a friend rather than a foe in these women^26,27^. The pathobiont *Gardnerella* was dominant in 9.8% of the Isala women. In previous studies, its association with symptoms and disease appeared to depend on the specific species and *s*trains, the other members in the vaginal community^28^ and the host^29^. In our cohort, the *Gardnerella* module was more associated with host health data (including HPV vaccination status) than *Gardnerella* as an individual taxon analysed. Hence, our modular approach indicates that an increased abundance of *Gardnerella* is not indicative of it being pathogenic per se, but rather reflective of different bacterial relationships and/or host states. This is a clear example that our module approach based on co-occurrence correlations between taxa could uncover some previously hidden associations. Yet, these modules will have to be further experimentally validated. One interpretation of these modules is that taxa within a module are positively metabolically or physically (for example, via co-aggregation) interacting with one or a few highly abundant ‘central’ taxa. Alternatively, interactions between the taxa could be indirect: taxa from the same module could be co-selected by a common host state or variable (for example, the local concentrations of specific hormones or sugars, pH, or HPV vaccination or other immunological status) or they could be co-dispersed from the same location (for example, the gut). While CSTs are helpful to reduce the complex high-dimensional nature of vaginal microbiome data^4,10^, it is unclear to what extent they conform to an underlying biological reality^30^. We observed here that many of our healthy samples lie in-between established CSTs, implying that abundant taxa co-occur relatively often, notably for *L. crispatus* and *L. iners*, in agreement with the previously suggested transitional states by Ravel and colleagues^9^. However, we did not find systematic co-occurrence between *L. crispatus* and *L. iners*. Rather, *L. crispatus* showed clear co-occurrence patterns with *L. jensenii* and *Limosilactobacillus*. This *L. crispatus* module probably reflects the most healthy homeostatic state based on our own observations of a reduced abundance of this module with increased numbers of vaginal complaints versus its increase with increasing oestrogen levels. This is in line with known associations of *L. crispatus* with vaginal health^2^. Notably, the association between this module and vaginal complaints was lost with the individual taxa, which suggests that the modules capture specific biological aspects better than individual taxa.

Another unprecedented finding for the *L. crispatus* module was the high prevalence when there was a low abundance of *Limosilactobacillus*. One possible explanation for these observations could be a keystone role for *Limosilactobacillus* taxa, which promotes *L. crispatus* and *L. jensenii* in their dominance in the vaginal niche. Positive interactions between different taxa of lactic acid bacteria are common in food fermentations where they dominate, for instance in yoghurt^31^ and kefir^32^_._ They have also been observed for related *Lactobacillus* taxa within vertebrate hosts, including the rodent gastrointestinal tract^33^. A widely used vaginal probiotic, *L. reuteri* RC-14, has been shown to prevent BV in women with human immunodeficiency virus (HIV)^34^ and improve the BV cure rate following a single dose of metronidazole when administered together with *Lacticaseibacillus rhamnosus* GR-1 (ref. ^35^). Further screening of *Limosilactobacillus* strains, not only from *L. reuteri* but also from other species of this genus such as *L. vaginalis* and *L. coleohomini*s, alone or in combination with other lactobacilli such as *L. crispatus* and *L. jensenii*, appears to be of interest for vaginal probiotic or live biotherapeutic product development.

The main motivation for our citizens to participate was to uncover associations between lifestyle and the vaginal microbiome derived from our co-created questionnaires. Thanks to our participants’ willingness to provide a rich source of survey data, we could confirm earlier established associations of the vaginal microbiome with BMI^36^, the contraceptive pill^37^ and smoking^38^. Notably, for contraceptive use, we could now even provide a higher-resolution association map, with the widely used oestrogen-containing contraceptives showing a positive and negative association with the levels of the *L. crispatus* and *Gardnerella* modules, respectively. While possible side effects such as negative impact on mood and libido^39,40^ and an increased risk of venous thromboembolism^41^ are increasingly highlighted, our data suggest a possible beneficial impact on the vaginal microbiome. On the other hand, women using a progestin-containing IUD had a significantly higher abundance of the *Gardnerella* module, in line with clinical data that insertion of a hormonal IUD temporarily increases BV and over time increases *Candida* spp. colonization in the vagina^42^.

A key finding of this study was that having been pregnant, including women that miscarried and/or had an abortion, was associated with the vaginal microbiome. Based on previous studies following pregnant women and women shortly after delivery, it was already documented that most women experience a decrease in *Lactobacillus* species and an increase in diverse anaerobes after delivery, which can persist for up to 1 year (ref. ^43^). Our citizen-science data show that the signature of a reduction in the *L. crispatus* module and an increase in taxa known to be among the first gut colonizers of babies (*Bifidobacterium*, *L. gasseri*, *Streptococcus* and *Alloscardovia*) is apparent in women having biological children. Hormonal and associated sugar-level changes during pregnancy (including lower oestrogens during breastfeeding), as well as the cervix shortening and vaginal dilatation during delivery versus C-section, could all be involved in this microbial shift and provide interesting angles for further research. Other intriguing associations we could uncover thanks to our citizens were related to sexual lifestyle and intimate partnership, such as women with a female partner being associated with more of the *L. crispatus* module and recent sexual intercourse associated with a higher frequency of the *Prevotella* module, as well as higher relative abundance of *Gemella*, *Staphylococcus*, *Streptococcus*, *Veillonella* and *Ureaplasma* among other taxa.

The associations found with dietary choices should be interpreted with care. Alcohol consumption, for example, was associated with a higher abundance of the *L. crispatus* module but has an established detrimental impact on the gut microbiome^44^ and many other aspects of health. By contrast, limiting intake of sugary drinks appears to be a lifestyle intervention that benefits multiple habitats that make up the human body. Dedicated longitudinal intervention studies with specific foods or diets, hygienic measures and/or probiotic species and strains should further substantiate the associations found and help the design of dedicated pharmaceutical and microbiome interventions, preferably co-created with citizens to further leverage the citizen-science approach. Finally, we highlight that by conscious communication employing appropriate tools and style, laywomen are eager to participate in taboo-breaking conversations as well as scientific studies aimed at improving their health. We therefore endorse citizen-science as a powerful approach to facilitate large-scale intimate microbiome research and to empower citizens to co-create research that impacts their individual and community-level health.

## Methods

### Study cohort and data collection

The study was approved by the Ethical Committee of the Antwerp University Hospital/University of Antwerp (B300201942076) and registered online at ClinicalTrials.gov with the unique identifier NCT04319536. The call for participants was launched on 24 March 2020, with the only inclusion criteria consisting of not being pregnant and being at least 18 years old. Within 10 days, 6,007 women registered through the Isala website (https://isala.be/en/) by answering five questions detailing age, postal code, previous pregnancies, residence country in their first 3 years and contraceptive use. After obtaining a digital informed consent, these participants were invited to fill out a large online survey with 139 questions that included relevant and General Data Protection Regulation-compliant questions on the Qualtrics platform (Qualtrics). The 4,682 participants who filled out the entire survey were invited to fill out their address on the website to receive an Isala self-sampling kit. Eventually, 4,106 self-sampling kits were sent out and 81.5% of the kits were returned to the University of Antwerp between July and October 2020. Two vaginal swabs were self-collected in a standardized way by non-pregnant participants (*n* = 3,323), and 3,294 participants filled out a short follow-up survey with 39 questions within 24 h of sampling.

Each kit contained two vaginal swabs. First the eNAT (Copan), intended for microbiome profiling, was collected and immediately afterwards the ESwab (Copan), intended for culturomics and metabolomics, was collected. Each self-sampling kit contained an elaborate brochure with infographics and instructions in layman terms. In addition, instruction videos on sampling, storage and transport were made and shared with participants. It was stipulated that both swabs had to be turned around two to three times to acquire enough biomass. Immediately after sampling, swabs were to be carefully transferred to a vial, which contained the commercial transport buffer of the eNAT or ESwab, and stored at home in the fridge. Finally, all samples were transported at ambient temperature (20–25 °C) with prepaid services by the national parcel service (Bpost) with an average transport time of 2.9 ± 3.3 days (*n* = 3,306) from which 92.8% arrived within 7 days from sampling. Upon arrival, the eNAT swabs were stored at −20 °C until further processing in the laboratory, which was optimized earlier^45^. The ESwab was vortexed for 15 s and separated in two aliquots of 500 µl, the first of which was stored at −80 °C in a 96 tube Micronic plate with 500 µl 50% glycerol, the other being centrifuged for 3 min at 13,000*g*, and its supernatant was stored in a 96 tube Micronic plate at −80 °C as well. All samples are stored in our decentral hub at the UZA/UAntwerpen Biobank. Samples are only available for secondary use after a Material Transfer Agreement, taking all General Data Protection Regulation and related regulations into account.

### 16S rRNA amplicon sequencing

Before further processing, all samples were vortexed for 15–30 s and extracted with the DNeasy PowerSoil Pro Kit, of which some were extracted manually and other extractions were automated with the QIAcube (Qiagen). DNA concentration of all samples was measured using the Qubit 3.0 Fluorometer (Life Technologies). No less than 2 µl of each bacterial DNA sample was used to amplify the V4 region of the 16S rRNA gene, using standard barcoded forward (515 F) and reverse (806 R) primers^45^. These primers were altered for dual-index paired-end sequencing, as described in Kozich et al.^46^. The resulting polymerase chain reaction (PCR) products were checked on a 1.2% agarose gel. The PCR products were then purified using the Agencourt AMPure XP Magnetic Bead Capture Kit (Beckman Coulter) and the concentration of all samples was measured using the Qubit 3.0 Fluorometer. Next, a library was prepared by pooling all PCR samples in equimolar concentrations. This library was loaded onto a 0.8% agarose gel and purified using the NucleoSpin Gel and PCR clean-up (Macherey-Nagel). The final concentration of the library was measured with the Qubit 3.0 Fluorometer. Afterwards the library was denatured with 0.2 N NaOH (Illumina), diluted to 6 pM and spiked with 10–15% PhiX control DNA (Illumina). Finally, dual-index paired-end sequencing was performed on a MiSeq Desktop sequencer (Illumina). All DNA samples, as well as controls of both PCR (PCR-grade water) and the DNA extraction runs, were included on the sequencing runs. In total, samples were sequenced across nine different MiSeq runs, with four to five extraction controls and three to four PCR controls per sequencing run.

### Construction of a custom 16S reference database

To increase taxonomic resolution for the genus *Lactobacillus*, three steps were defined to split this genus into nine subgenera. First, a maximum-likelihood species phylogeny of the genus was constructed using amino acid sequences of 100 single-copy core genes (selected to minimize gene absence) from representative genomes, using the software IQ-TREE^47^. Second, the subgenera were manually defined as the minimum number of clades in the species phylogeny that would be needed to discriminate the four major vaginal *Lactobacillus* species. Finally, the subgenera were checked for monophyly against the species phylogeny of release 05-RS95 (Supplementary Fig. 1) of the Genome Taxonomy Database (GTDB)^48^.

To classify amplicon sequences to the *Lactobacillus* subgenera, a custom 16S rRNA reference database was created by downloading 16S rRNA sequences from sequenced genomes from the GTDB (release 05-RS95), as well as from the GTDB taxonomy hierarchy. This dataset was reduced to sequences of the family *Lactobacillaceae* only, and the genus *Lactobacillus* in the taxonomy hierarchy was replaced by the respective subgenera of the species. Finally, these files were converted into a DADA2-compatible reference database.

### Processing and quality control of amplicon sequencing data

Quality control and processing of amplicon reads was performed with the R package DADA2, version 1.22.0 (ref. ^49^). First, reads with more than two expected errors were removed (no trimming was performed). Next, paired reads were merged; in this process, read pairs with one or more sequence conflicts were removed. Chimeras were detected and removed with the removeBimeraDenovo function. The merged and denoised reads (ASVs) were taxonomically annotated from the phylum to the genus level with the assignTaxonomy function using the EzBioCloud reference 16S rRNA database^50^. Next, three different reclassifications were performed. First, ASVs classified to the family Leuconostocaceae were reclassified to the family Lactobacillaceae to be in line with the recent taxonomic update^5^. Second, the Lactobacillaceae ASVs were reclassified on the genus level to the new genera defined by Zheng et al.^5^. And finally, ASVs of the updated genus *Lactobacillus* (previously known as the *Lactobacillus delbrueckii* group) were reclassified into nine different subgenera that we manually defined on the basis of the phylogeny of the genus.

Taxon and sample quality control was performed as follows. Non-bacterial ASVs (for example, mitochondria) and ASVs with a length greater than 260 bases were removed. Quality control of the samples was based on normalized read concentrations, which were calculated as follows. First, the total read count per sample was divided by the volume of that sample added to its respective MiSeq sequencing run (there were nine runs in total). Next, these read concentrations were normalized by dividing them by the median read concentration of their respective run. Samples were then filtered using two criteria: (1) the normalized read concentration should be higher than 0.05, a cut-off chosen based on a manual inspection of the negative controls (Supplementary Fig. 17), and (2) the read count of a sample should be greater than 2,000.

### Validation of the amplicon sequencing pipeline including *Lactobacillus* subclassification

To validate our amplicon sequencing pipeline, including *Lactobacillus* subgenus classification, we used a dataset of samples that were sequenced with both amplicon (V4 region of the 16S rRNA gene) and metagenome sequencing for the Isala pilot study^45^. DNA was extracted with the HostZERO Microbial DNA Kit (Zymo Research).

To be able to directly compare the results of both sequencing techniques, we constructed amplicon and metagenome taxonomic reference databases that use a consistent taxonomy. For the amplicon samples, we downloaded the 16S sequences of version 05-RS95 of the GTDB and changed the *Lactobacillus* genus labels to the nine custom subgenera that we defined. For the metagenome samples, we created a reference database using three pieces of data: (1) representative genomes for all bacterial species, downloaded from release 05-RS95 of the GTDB, (2) the GTDB taxonomy hierarchy updated with the *Lactobacillus* subgenera and (3) version 38 of the human genome of the Genome Reference Consortium (GRCh38), downloaded from NCBI RefSeq^51^. These files were used to create a database in Kraken2-compatible format.

The amplicon samples from the Isala pilot study were processed in the same way as the amplicon samples of this study, except that reads were classified using our custom GTDB-based reference database and that sample quality control was based on a minimum read count of 5,000 reads. Metagenomic shotgun sequenced samples from the Isala pilot study were processed as follows. First, paired reads were filtered with the DADA2 R package, version 1.20.0 (ref. ^49^), requiring a minimum length of 50 bases, a maximum of two uncalled bases per read and a maximum of two expected errors per read. Next, read pairs were classified from the phylum to the species level with Kraken2 (ref. ^52^), using our custom GTDB-based reference database. Non-bacterial taxa were removed from the data, as were samples with fewer than 15,000 bacterial reads. After sample processing and quality control, 18 pairs of samples remained where at least one high-quality amplicon and metagenome samples were available from the same participant.

To assess whether our pipeline was able to differentiate between V4 reads of the *L. crispatus* group, *L. iners* group, *L. gasseri* group and *L. jensenii* group, we compared the relative abundance vector of each of these subgenera between the amplicon and shotgun samples by calculating the Spearman correlation.

As a second independent validation of the subgenus-level classification of V4 reads, we classified V4 sequences extracted from 1,121 whole genomes of *Lactobacillus* that we downloaded from NCBI GenBank. After removing seven unique V4 sequences that we identified as contaminants because they occurred only in a single genome and showed high similarity to 16S sequences from bacteria unrelated to *Lactobacillus*, the known subgenus of the remaining sequences was compared with the subgenus predicted by our amplicon pipeline.

### Contraceptives, menstrual cycle and hormonal levels

Upon sampling, participants indicated when their menstrual cycle began, and the average length of their cycle. Depending upon the contraceptive method, these data were used to determine the day in the cycle and predict the endogenous and exogenous levels of oestrogen and progestin. Peri- and post-menopausal women were excluded from this analysis.

### Additional datasets

The VALENCIA dataset was sourced from GitHub^53^. Data from the Human Microbiome Project (VaHMP) were downloaded from https://portal.hmpdacc.org/ using the query ‘sample.study_name in [‘MOMS-PI’,‘HHS’,‘16S-VM-DGE’] and file.node_type in [‘abundance_matrix’] and sample.body_site in [‘vagina’] and file.format in [‘Biological Observation Matrix’]’. For downstream analysis and comparing with other datasets, abundance data were aggregated at the genus level, except for *L. crispatus*, *L. iners*, *L. jensenii* and *L. gasseri*.

### CST mapping

Mapping of Isala samples to the VALENCIA CSTs was done by manually matching taxa between the Isala taxonomy and the VALENCIA taxonomy^10^ and running the VALENCIA CST mapping tool with on the Isala data with manually adapted taxon names.

### Embeddings

Principal coordinates analysis, uniform manifold approximation and projection, and *t*-SNE-embeddings were performed on the relative (sub)genus abundances per sample, using the Bray–Curtis distance metric^54^ to calculate distances within the *t*-SNE^20^. Samples were labelled with the first or second most dominant taxa, except if that taxon occurred less than 200 times as the most dominant taxon, in which case it was labelled as ‘other’.

### Statistical analyses

To determine correlations between the abundances of taxa across our samples, we used the SparCC^21^ as implemented in the spiec-easi^55^ package with 1,000 permutations. We calculated correlations only between taxa that were present (abundance higher than zero) in at least 10% of all samples^21^. Clusters of taxa were identified with MCL^56^ with default settings as implemented in the MCL R package. Validating the clusters found in the Isala network was done by examining the correlation networks identified in the VALENCIA and VaHMP datasets and manually confirming that the correlations identified within each module were also identified in the Isala data and were also present in at least one other dataset (Supplementary Fig. 11). Networks for VALENCIA and VaHMP were calculated using a single sample per participant. Furthermore, correlations between the networks in the three datasets were tested with a Mantel test with 10,000 permutations. Eigentaxa, a summary score for a given set of taxa (determined by the modules identified in the taxa-taxa correlation networks), were calculated first by centred log ratio (CLR) transforming the relative abundance data and taking the first principal component of the taxa in each cluster^57^. Eigentaxa were multiplied by the sign of the correlation coefficient between the eigentaxa and the most highly abundant taxon for each cluster.

No statistical methods were used to pre-determine sample sizes, but the number of participants was much larger than previous large-scale studies on the vaginal microbiome (VALENCIA^10^ and VaHMP^13^). Associations between microbial community composition (beta diversity) and the survey data (Supplementary Table 5 and Supplementary Text 1) were performed with an Adonis test, as implemented in the vegan package in R (ref. ^58^). For computational efficiency, the adonis2 function was modified to perform permutations only for the variable of interest and with the parameter by=’margin’. For each effect of interest, we tested the following model: ~ e_i + age + antibiotic + intercourse + e_t, where e_t are technical effects, e_i is the effect of interest, antibiotic is the use of antibiotics in the past 3 months and intercourse is whether intercourse took place in the past 24 h. Technical effects used were identical across all experiments and consisted of sequencing run, normalized read concentration and library size, which were found to be strongly associated with the principal components of the relative abundance. To optimize computational performance, initially 1,000 permutations were performed for each effect of interest. A total of 10,000 permutations were performed only for those effects that had *P* values equal and smaller than 0.001. Multiple testing correction was done with the Benjamini–Hochberg procedure^59^.

Associations between Shannon diversity and our factors collected via the survey were performed with a multiple linear regression, with the same models used in the Adonis test. Diversity values were *z*-standardized before testing. Multiple testing correction was done with the Benjamini–Hochberg procedure.

Associations between the assigned VALENCIA CSTs and our factors were performed with a one-versus-all logistic regression with the same models used in the Adonis test. Associations between the factors and the module eigentaxa were evaluated with a linear regression. Eigentaxa values were *z*-standardized before testing. Multiple testing correction was done with the Benjamini–Hochberg procedure, adjusting across all modules/CSTs and factors^60^.

As differential abundance tools often find different results^23^, we implemented six differential abundance tools that can adjust for covariates into a single R package: multidiffabundance^61^. Hereto, ALDeX2 (ref. ^62^), ANCOM-BC^63^, DESeq2 (ref. ^64^), limma^65^, a linear regression on the CLR-transformed abundance data, and Maaslin2 (ref. ^66^) were used. Only taxa that were present in at least 10% of all samples were tested. Multiple testing correction was done with the Benjamini–Hochberg procedure, across all factors and taxa (we did not adjust across the number of methods tested). Each differential abundance test assumes a different distribution of taxa abundances, but this was not formally tested. All data handling and visualization was performed in Python 3 and R 4 (ref. ^67^) using the tidyverse set of packages and the in-house developed package tidyamplicons^68^.

### Questionnaire design

This survey was set up in collaboration with input from experts (mainly from team Prof. Lebeer for microbiome-related parameters, Prof. De Backer for communication- and food-related questions, Dr Masquiller for sociology-related questions, Sensoa for sexual lifestyle-related questions, Provincial Institute for Hygiene for general health-related questions and Prof. K. Scott for food-related questions) and non-experts (citizens) in the context of Flanders in the year 2020. Questions were asked in a standardized way when possible via the Qualtrics platform, but always taking the ‘citizen-science aspect’ of the project into account. The study was not set up as a population study, but as a citizen-science project. It reflects a specific geography and zeitgeist. The full questionnaire can be found in Supplementary Text 1.

### Reporting summary

Further information on research design is available in the Nature Portfolio Reporting Summary linked to this article.

### Supplementary information

Supplementary Information Supplementary Figs. 1–17 and Text 1. Also available on figshare at https://doi.org/10.6084/m9.figshare.23099480.

Reporting summary

Supplementary Tables 1–11 Supplementary Table 1. Descriptive statistics of taxa. Various descriptive statistics for subgenera of the genus *Lactobacillus* and genera detected in this study: number of ASVs within the (sub)genus (n_asvs), prevalence (occurrence), average relative abundance (mean_rel_abundance), frequency of being the most abundant taxon and greater than 0% abundant (top_and_gt0p), same as previous but greater than 30% abundant (top_and_gt30p), same as previous but greater than 50% abundant (top_and_gt50p), and the previous three measures but in terms of relative frequencies (top_and_gtXp_rel). Also available on Figshare: https://doi.org/10.6084/m9.figshare.23100350. Table 2. Table of the number of Isala participants per VALENCIA subCST. Table 3. Taxa–taxa correlation network generated from SparCC for the Isala dataset. Each cell indicates the compositionality aware correlation between two taxa. Table 4. Association tests between participant characteristics and their vaginal microbiome. Results of statistical tests for each tested questionnaire response. Results of association tests are provided for beta diversity (Adonis tests), alpha diversity, CSTs, eigentaxa and individual taxa. In addition to effect sizes, test statistics and *P* values, the number of participants in each condition is provided. Table 5. Supplementary meta-data for the Isala samples used in this study. Each European Nucleotide Archive sample ID is linked to a participant’s age, whether intercourse occurred in the past 24 h, technical covariates and CST annotations. This file can be used in combination with the code available on GitHub. Table 6. Results of the permutational multivariate analysis of variance. Adonis2 tests between technical factors and the vaginal microbiome. Table 7. Count data per (sub)genus per sample. Linked by identified to the meta-data provided on EGA and Supplementary Table 5. This file can be used in combination with the code available on GitHub. Table 8. Relative abundance data per sample. Linked by and identified to the meta-data provided on EGA and Supplementary Table 5. Table 9. Taxa classification specification per (sub)genus. Specified in Supplementary Tables 7 and 8. This file can be used in combination with the code available on GitHub. Table 10. Count data per ASV per sample. Linked by and identified to the meta-data provided on EGA and Supplementary Table 3. This file can be used in combination with the code available on GitHub. Table 11. Taxa classification specification per ASV. Specified in Supplementary Table 10. This file can be used in combination with the code available on GitHub.

## Data Availability

Sequencing data are available at the European Nucleotide Archive under bioproject PRJEB50407. Sample meta-data are available with access control via the European Genome–Phenome Archive (EGA) under dataset ID EGAD00001009890, where data can be accessed as described upon agreeing to the harmonised Data Access Agreement (developed by the European Union standards for in silico models for personalized medicine EU-STANDS4PM, 10.6084/m9.figshare.23904300). A guide to data access on the EGA is available at https://ega-archive.org/access/data-access, and a data access request will be processed within 2–3 months, pending evaluation by the data access committee, and processing by the EGA. A description of all meta-data is available on Figshare at 10.6084/m9.figshare.23904300). A minimal meta-data table can be found in Supplementary Table 3, genus-level count data per sample can be found in Supplementary Table 7, genus-level relative abundance data can be found in Supplementary Table 8, a genus-level taxa specification table can be found in Supplementary Table 9, an ASV level count table can be found in Supplementary Table 10 and an ASV level taxa specification can be found in Supplementary Table 11. All supplementary tables are also available on figshare at 10.6084/m9.figshare.23100350. All swabs are stored in our local biobank and available for secondary use upon a material transfer agreement and mutual agreement, according to our Belgian law respecting the informed consent.

## Code availability

The in-house amplicon data processing toolkit used is available at https://github.com/SWittouck/tidyamplicons. The tool to perform differential abundance with multiple different testing tools is available at https://github.com/thiesgehrmann/multidiffabundance. All other scripts used for data processing and analysis are hosted on GitHub and accessible at https://github.com/LebeerLab/Citizen-science-map-of-the-vaginal-microbiome.

## References

[1] Lash AF, Kaplan B (1928). A study of Döderlein’ s vaginal bacillus. Oxford Univ. Press.

[2] Petrova MI, Lievens E, Malik S, Imholz N, Lebeer S (2015). *Lactobacillus* species as biomarkers and agents that can promote various aspects of vaginal health. Front. Physiol..

[3] Younes JA (2018). Women and their microbes: the unexpected friendship. Trends Microbiol..

[4] Ravel J (2011). Vaginal microbiome of reproductive-age women. Proc. Natl Acad. Sci. USA.

[5] Zheng J (2020). A taxonomic note on the genus *Lactobacillus*: description of 23 novel genera, emended description of the genus *Lactobacillus beijerinck* 1901, and union of Lactobacillaceae and Leuconostocaceae. Int. J. Syst. Evol. Microbiol..

[6] Vaneechoutte M (2019). Emended description of *Gardnerella vaginalis* and description of *Gardnerella leopoldii* sp. nov., *Gardnerella piotii* sp. nov. and *Gardnerella swidsinskii* sp. nov., with delineation of 13 genomic species within the genus *Gardnerella*. Int. J. Syst. Evol. Microbiol..

[7] Nouioui I (2018). Genome-based taxonomic classification of the phylum Actinobacteria. Front. Microbiol..

[8] Hitch TCA (2022). A taxonomic note on the genus *Prevotella*: description of four novel genera and emended description of the genera *Hallella* and *Xylanibacter*. Syst. Appl. Microbiol..

[9] Gajer P (2012). Temporal dynamics of the human vaginal microbiota. Sci. Transl. Med..

[10] France M (2020). VALENCIA: a nearest centroid classification method for vaginal microbial communities based on composition. Microbiome.

[11] Miller EA, Beasley DAE, Dunn RR, Archie EA (2016). Lactobacilli dominance and vaginal pH: why is the human vaginal microbiome unique?. Front. Microbiol..

[12] Condori S (2022). Recent insights into the vaginal microbiota. Microbiota Heal. Dis..

[13] Serrano MG (2019). Racioethnic diversity in the dynamics of the vaginal microbiome during pregnancy. Nat. Med..

[14] Drell T (2013). Characterization of the vaginal micro- and mycobiome in asymptomatic reproductive-age estonian women. PLoS ONE.

[15] Lennard K (2017). Microbial composition predicts genital tract inflammation and persistent bacterial vaginosis in South African adolescent females. Infect. Immun..

[16] Mehta, S. D., Nannini, D. R. & Otieno, F. Host genetic factors associated with vaginal microbiome. *mSystems* **8**, e00502–e00520 (2020). 10.1128/mSystems.00502-20 PMC7394359 32723796

[17] Rivera-Riquelme M, Piqueras JA, Cuijpers P (2019). The Revised Mental Health Inventory-5 (MHI-5) as an ultra-brief screening measure of bidimensional mental health in children and adolescents. Psychiatry Res..

[18] Ost C, De Ridder KAA, Tafforeau J, Oyen H (2017). The added value of food frequency questionnaire (FFQ) information to estimate the usual food intake based on repeated 24-hour recalls. Arch. Public Heal..

[19] Ma B (2020). A comprehensive non-redundant gene catalog reveals extensive within-community intraspecies diversity in the human vagina. Nat. Commun..

[20] van der Maaten L, Hinton G (2008). Visualizing data using *t*-SNE. J. Mach. Learn. Res..

[21] Watts SC, Ritchie SC, Inouye M, Holt KE (2019). FastSpar: rapid and scalable correlation estimation for compositional data. Bioinformatics.

[22] Enright AJ, Van Dongen S, Ouzounis CA (2002). An efficient algorithm for large-scale detection of protein families. Nucleic Acids Res..

[23] Nearing, J. T. et al. Microbiome differential abundance methods produce different results across 38 datasets. *Nat. Commun.* **13**, 342 (2022). 10.1038/s41467-022-28034-z PMC8763921 35039521

[24] Falony G (2016). Population-level analysis of gut microbiome variation. Science.

[25] Crespo, B. V. et al. Role of self-sampling for cervical cancer screening: diagnostic test properties of three tests for the diagnosis of HPV in rural communities of Cuenca, Ecuador. *Int. J. Environ. Res. Public Health* **19**, 4619 (2022). 10.3390/ijerph19084619 PMC9028024 35457487

[26] Petrova MI, Reid G, Vaneechoutte M, Lebeer S (2017). *Lactobacillus iners*: friend or foe?. Trends Microbiol..

[27] France MT (2020). Complete genome sequences of six *Lactobacillus iners* strains isolated from the human vagina. Microbiol. Resour. Announc..

[28] Łaniewski, P. & Herbst-Kralovetz, M. M. Bacterial vaginosis and health-associated bacteria modulate the immunometabolic landscape in 3D model of human cervix. *npj Biofilms Microbiomes* **7**, 88 (2021). 10.1038/s41522-021-00259-8 PMC8669023 34903740

[29] Castro J, Jefferson KK, Cerca N (2020). Genetic heterogeneity and taxonomic diversity among *Gardnerella* species. Trends Microbiol..

[30] Charbonneau MR (2016). A microbial perspective of human developmental biology. Nature.

[31] Canon, F., Nidelet, T., Guédon, E., Thierry, A. & Gagnaire, V. Understanding the mechanisms of positive microbial interactions that benefit lactic acid bacteria co-cultures. *Front. Microbiol.* **11**, 2088 (2020). 10.3389/fmicb.2020.02088 PMC7500094 33013761

[32] Blasche S (2021). Metabolic cooperation and spatiotemporal niche partitioning in a kefir microbial community. Nat. Microbiol..

[33] Lin XB (2018). The evolution of ecological facilitation within mixed-species biofilms in the mouse gastrointestinal tract. ISME J..

[34] Hummelen R (2010). *Lactobacillus rhamnosus* GR-1 and *L. reuteri* RC-14 to prevent or cure bacterial vaginosis among women with HIV. Int. J. Gynecol. Obstet..

[35] Martinez RCR (2009). Improved cure of bacterial vaginosis with single dose of tinidazole (2 g), *Lactobacillus rhamnosus* GR-1, and *Lactobacillus reuteri* RC-14: a randomized, double-blind, placebo-controlled trial. Can. J. Microbiol..

[36] Si J, You HJ, Yu J, Sung J, Ko GP (2017). *Prevotella* as a hub for vaginal microbiota under the influence of host genetics and their association with obesity. Cell Host Microbe.

[37] Vodstrcil, L. A. et al. Combined oral contraceptive pill-exposure alone does not reduce the risk of bacterial vaginosis recurrence in a pilot randomised controlled trial. *Sci. Rep.* **9**, 3555 (2019). 10.1038/s41598-019-39879-8 PMC6401172 30837554

[38] Nelson TM (2018). Cigarette smoking is associated with an altered vaginal tract metabolomic profile. Sci. Rep..

[39] Lewis CA (2019). Effects of hormonal contraceptives on mood: a focus on emotion recognition and reactivity, reward processing, and stress response. Curr. Psychiatry Rep..

[40] Burrows LJ, Basha M, Goldstein AT (2012). The effects of hormonal contraceptives on female sexuality: a review. J. Sex. Med..

[41] Morimont L, Haguet H, Dogné JM, Gaspard U, Douxfils J (2021). Combined oral contraceptives and venous thromboembolism: review and perspective to mitigate the risk. Front. Endocrinol..

[42] Donders GGG (2016). Screening for abnormal vaginal microflora by self-assessed vaginal pH does not enable detection of sexually transmitted infections in Ugandan women. Diagn. Microbiol. Infect. Dis..

[43] DiGiulio DB (2015). Temporal and spatial variation of the human microbiota during pregnancy. Proc. Natl Acad. Sci. USA.

[44] Lee E, Lee JE (2021). Impact of drinking alcohol on gut microbiota: recent perspectives on ethanol and alcoholic beverage. Curr. Opin. Food Sci..

[45] Ahannach S (2021). Microbial enrichment and storage for metagenomics of vaginal, skin, and saliva samples. iScience.

[46] Kozich JJ, Westcott SL, Baxter NT, Highlander SK, Schloss PD (2013). Development of a dual-index sequencing strategy and curation pipeline for analyzing amplicon sequence data on the MiSeq Illumina sequencing platform. Appl. Environ. Microbiol..

[47] Nguyen LT, Schmidt HA, Von Haeseler A, Minh BQ (2015). IQ-TREE: a fast and effective stochastic algorithm for estimating maximum-likelihood phylogenies. Mol. Biol. Evol..

[48] Parks, D. H. et al. GTDB: an ongoing census of bacterial and archaeal diversity through a phylogenetically consistent, rank normalized and complete genome-based taxonomy. *Nucleic Acids Res.* **50**, D785–D794 (2022). 10.1093/nar/gkab776 PMC8728215 34520557

[49] Callahan BJ (2016). DADA2: high-resolution sample inference from Illumina amplicon data. Nat. Methods.

[50] Yoon SH (2017). Introducing EzBioCloud: a taxonomically united database of 16S rRNA gene sequences and whole-genome assemblies. Int. J. Syst. Evol. Microbiol..

[51] O’Leary NA (2016). Reference sequence (RefSeq) database at NCBI: current status, taxonomic expansion, and functional annotation. Nucleic Acids Res..

[52] Wood DE, Salzberg SL (2014). Kraken: ultrafast metagenomic sequence classification using exact alignments. Genome Biol..

[53] VALENCIA. *GitHub* https://github.com/ravel-lab/VALENCIA/ (2023).

[54] van der Maaten, L. Accelerating t-SNE using tree-based algorithms. *J. Mach. Learn. Res.* **15**, 3221–3245 (2014).

[55] Kurtz, Z. D. et al. Sparse and compositionally robust inference of microbial ecological networks. *PLoS Comput. Biol.* **11**, e1004226 (2015). 10.1371/journal.pcbi.1004226 PMC4423992 25950956

[56] Van Dongen, S. *MCL*—*A Cluster Algorithm for Graphs* (CWI, 1998).

[57] Caslin B (2019). Alcohol shifts gut microbial networks and ameliorates a murine model of neuroinflammation in a sex-specific pattern. Proc. Natl Acad. Sci. USA.

[58] Oksanen, J. A. R. I. et al. vegan: community ecology package (R package version 2.5–7). *GitHub* https://github.com/vegandevs/vegan (2022).

[59] Benjamini Y, Hochberg Y (1995). Controlling the false discovery rate: a practical and powerful approach to multiple testing. J. R. Stat. Soc..

[60] Aitchison, J. A concise guide to compositional data analysis. in *2nd Compositional Data Analysis Workshop* (University of Girona, 2005).

[61] multidiffabundance. *GitHub* https://github.com/thiesgehrmann/multidiffabundance (2023).

[62] Anders S (2013). Count-based differential expression analysis of RNA sequencing data using R and Bioconductor. Nat. Protoc..

[63] Lin, H. & Peddada, S. Das Analysis of compositions of microbiomes with bias correction. *Nat. Commun.* **11**, 3514 (2020). 10.1038/s41467-020-17041-7 PMC7360769 32665548

[64] Love, M. I., Huber, W. & Anders, S. Moderated estimation of fold change and dispersion for RNA-seq data with DESeq2. *Genome Biol.* **15**, 550 (2014). 10.1186/s13059-014-0550-8 PMC4302049 25516281

[65] Law, C. W., Chen, Y., Shi, W. & Smyth, G. K. voom: precision weights unlock linear model analysis tools for RNA-seq read counts. *Genome Biol.* **15**, R29 (2014). 10.1186/gb-2014-15-2-r29 PMC4053721 24485249

[66] Mallick, H. et al. Multivariable association discovery in population-scale meta-omics studies. *PLoS Comput. Biol.* **17**, e1009442 (2021). 10.1371/journal.pcbi.1009442 PMC8714082 34784344

[67] R Core Team. *R: A Language and Environment for Statistical Computing* (R Foundation for Statistical Computing, 2020).

[68] tidyamplicons. *GitHub* github.com/Swittouck/tidyamplicons (2021).

[69] Rocha J (2020). *Lactobacillus mulieris* sp. nov., a new species of *Lactobacillus delbrueckii* group. Int. J. Syst. Evol. Microbiol..

