# Supplementary material for: A citizen-science-enabled catalogue of the vaginal microbiome and associated factors
Source: Nat Microbiol. 2023 Oct 26;8(11):2183–95. doi: 10.1038/s41564-023-01500-0 (PMC10627828; doi:10.1038/s41564-023-01500-0)
Supplement: Supplementary file 1 — Supplementary Figs. 1–17 and Text 1. Also available on figshare at https://doi.org/10.6084/m9.figshare.23099480. [file 41564_2023_1500_MOESM1_ESM.pdf]

nature microbiology

Resource

https://doi.org/10.1038/s41564-023-01500-0

A citizen-science-enabled catalogue of the vaginal microbiome and associated factors

In the format provided by the authors and unedited

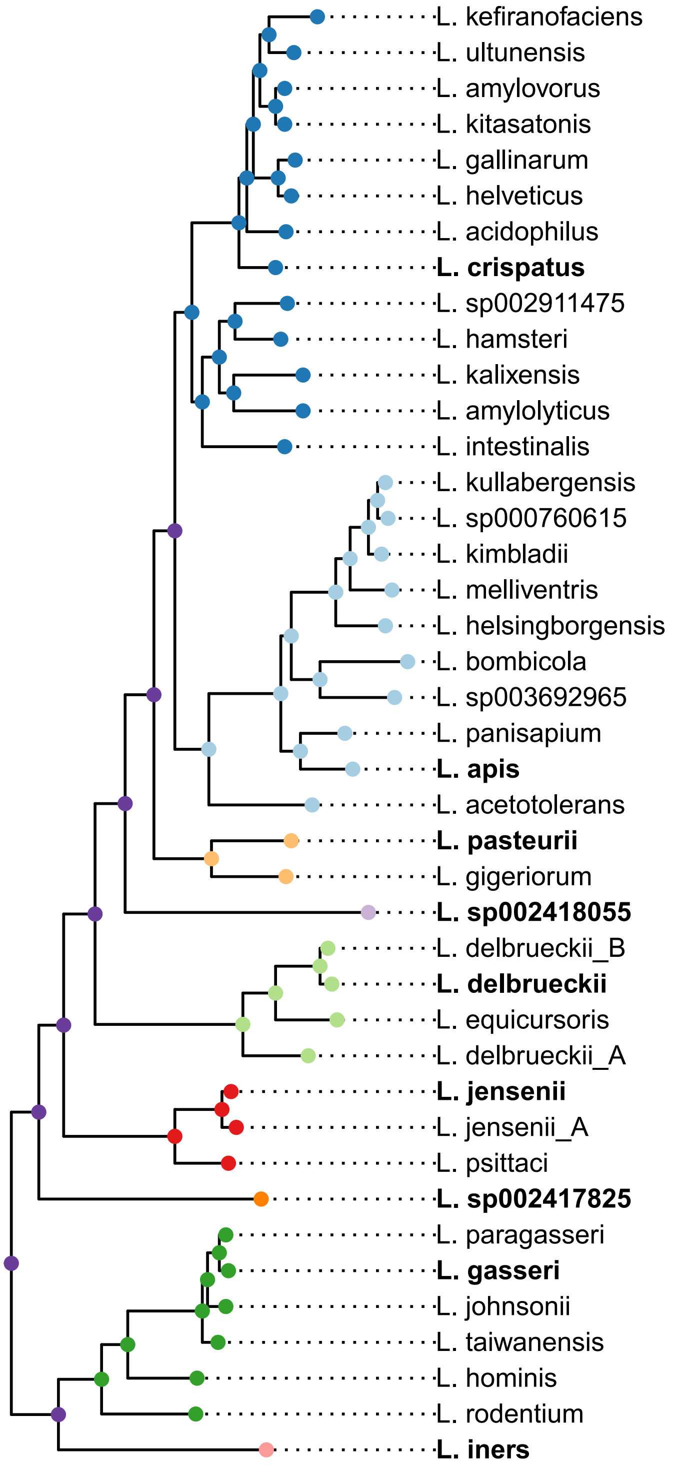

Supplementary Figure 1 - Species tree of the genus *Lactobacillus* from the Genome Taxonomy Database. Maximum-likelihood species phylogeny of the genus *Lactobacillus*, obtained by taking a subtree of the species phylogeny of the domain Bacteria inferred by the Genome Taxonomy Database (GTDB), release 05-RS95[46]. Colors indicate the nine custom-defined subgenera used in this study. Bold tip labels indicate representative species of the subgenera.

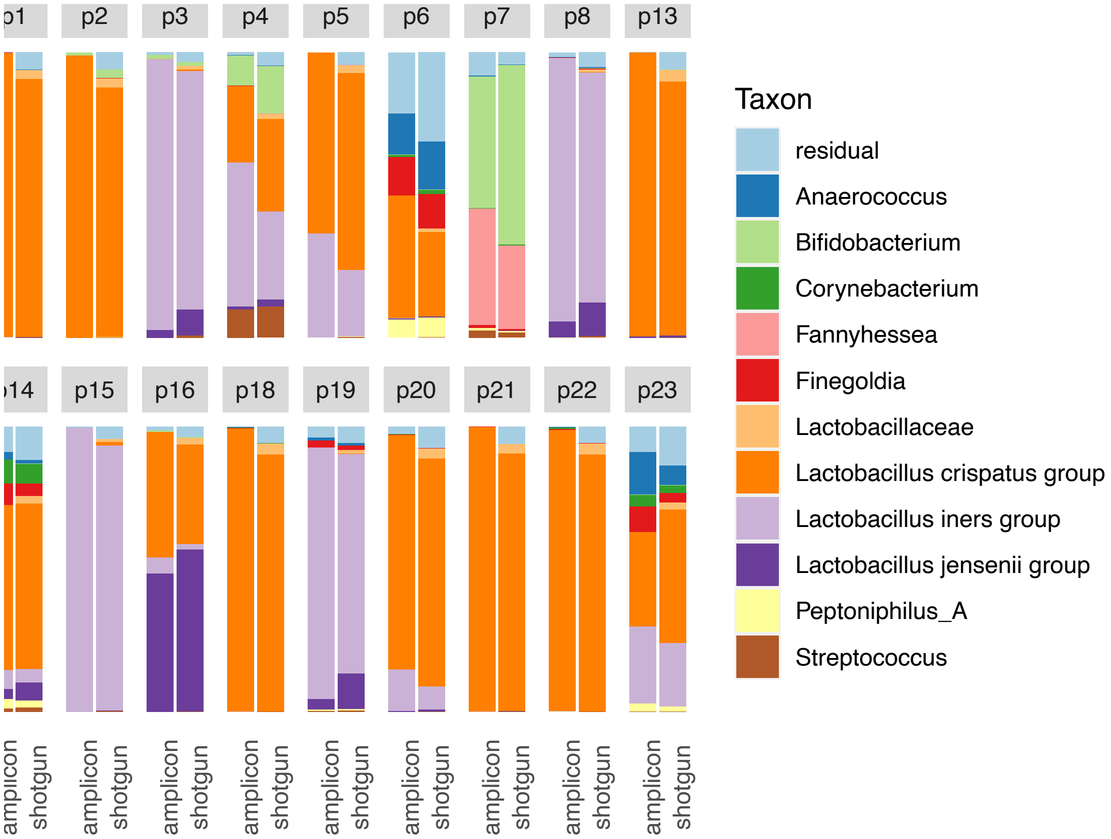

Supplementary Figure 2 - Comparison between amplicon and metagenome sequencing results for 18 samples. Relative abundances for the eleven most abundant taxa overall. Each facet shows a vaginal sample from a single participant, sequenced with 16S rRNA amplicon sequencing (left) or metagenomic shotgun sequencing (right).

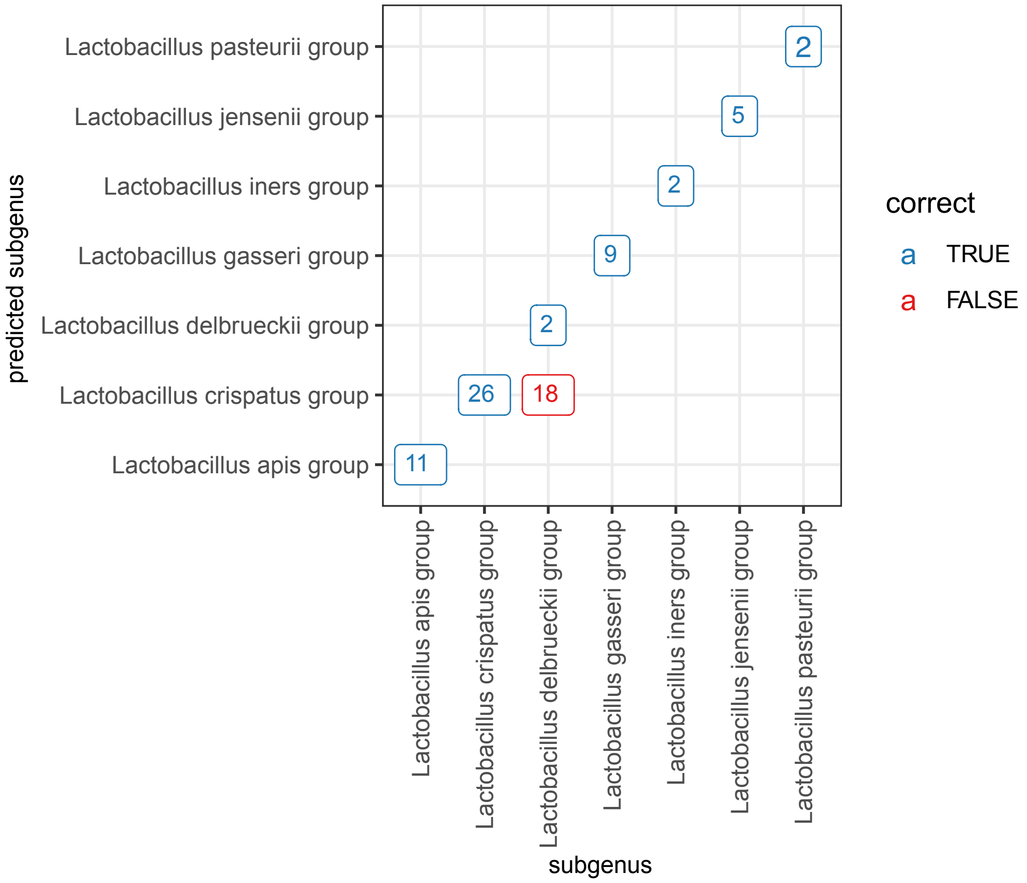

Supplementary Figure 3 – Validation of Lactobacillus subgenus classification of V4 sequences. Numbers of unique ASV sequences extracted from 1,121 public Lactobacillus genomes. The true subgenus (as deduced from the GTDB species labels of the genomes that the V4 sequence is present in) is represented on the x-axis. The subgenus assigned by our V4 classification pipeline (including database with *Lactobacillus* subgenera) is represented on the y-axis.

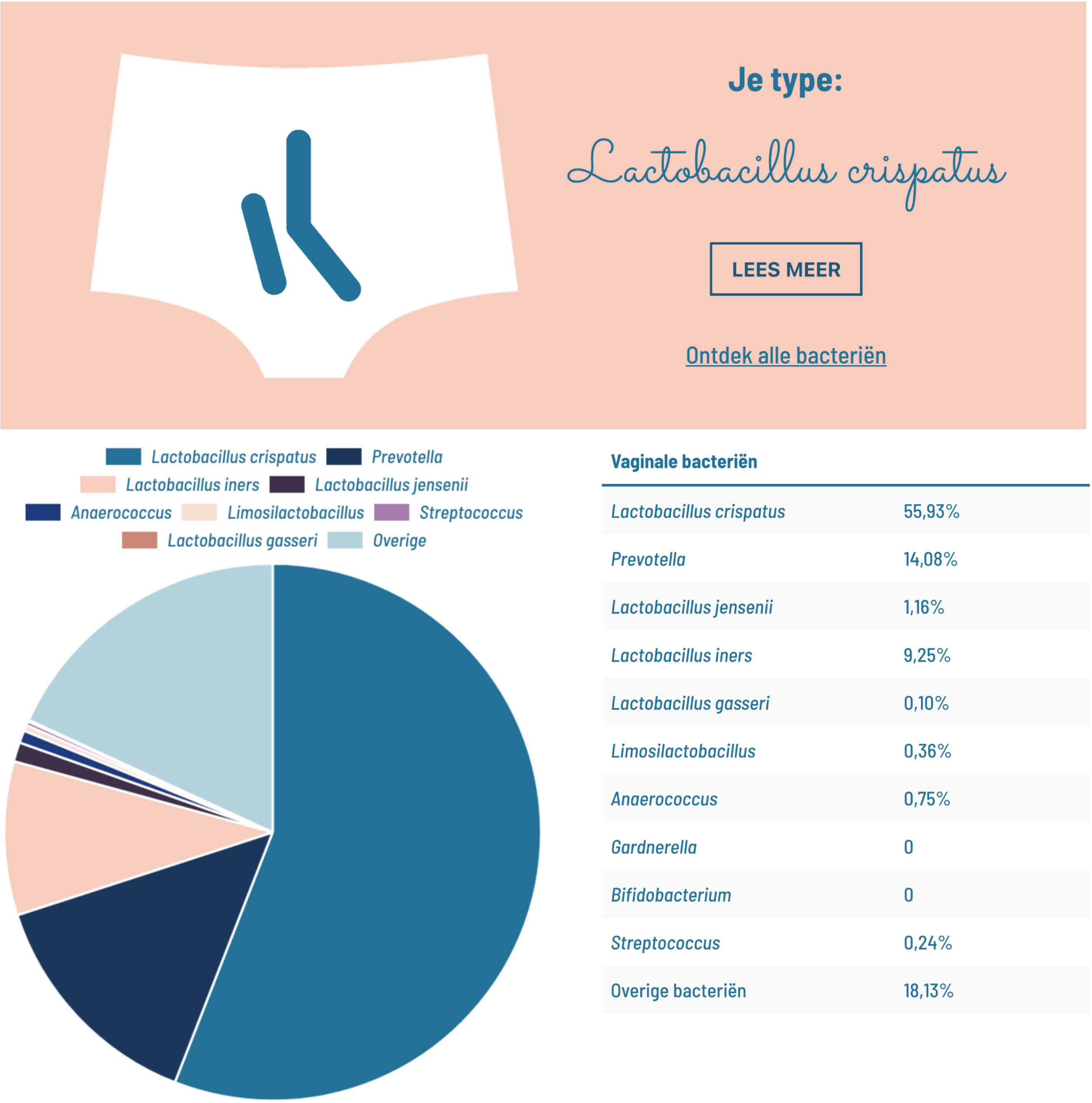

Vaginale bacteriën	
Lactobacillus crispatus	55,93%
Prevotella	14,08%
Lactobacillus jensenii	1,16%
Lactobacillus iners	9,25%
Lactobacillus gasseri	0,10%
Limosilactobacillus	0,36%
Anaerococcus	0,75%
Gardnerella	0
Bifidobacterium	0
Streptococcus	0,24%
Overige bacteriën	18,13%

Supplementary Figure 4 - Example of a personal vaginal microbiome profile result. Top left figure indicates the dominant (sub)genus. Bottom left shows a pie chart with the relative abundance of the top eight taxa identified in the sample. The table on the bottom right lists the relative abundance of the ten overall most abundant taxa in the entire cohort plus the percentage of the remaining taxa ("Overige bacteriën").

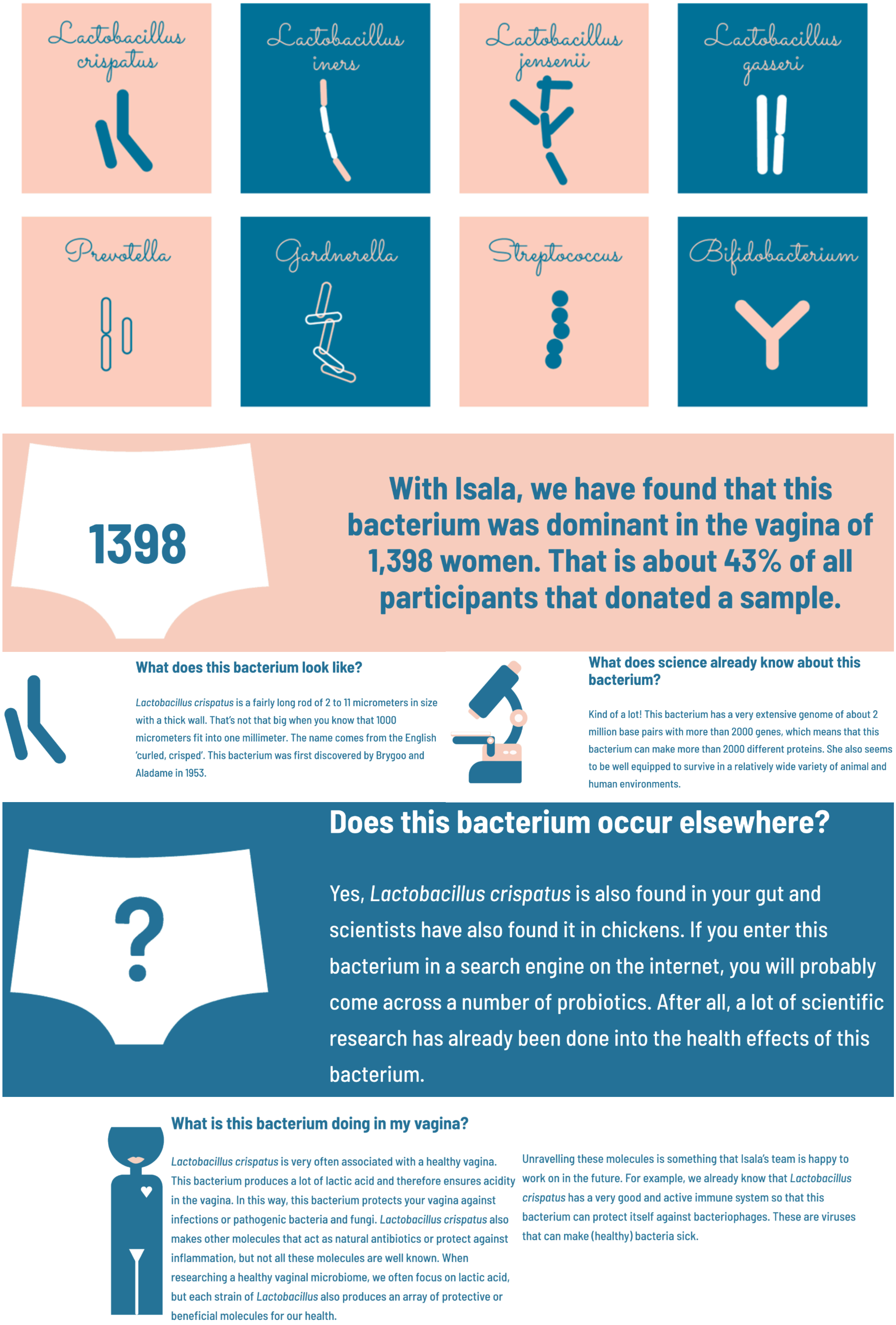
Lactobacillus crispatus
Lactobacillus iners
Lactobacillus jensenii
Lactobacillus gasseri
Prevotella
Gardnerella
Streptococcus
Bifidobacterium

1398

With Isala, we have found that this bacterium was dominant in the vagina of 1,398 women. That is about 43% of all participants that donated a sample.

What does this bacterium look like?

Lactobacillus crispatus is a fairly long rod of 2 to 11 micrometers in size with a thick wall. That's not that big when you know that 1000 micrometers fit into one millimeter. The name comes from the English 'curled, crisped'. This bacterium was first discovered by Brygoo and Aladame in 1953.

What does science already know about this bacterium?

Kind of a lot! This bacterium has a very extensive genome of about 2 million base pairs with more than 2000 genes, which means that this bacterium can make more than 2000 different proteins. She also seems to be well equipped to survive in a relatively wide variety of animal and human environments.

?

Does this bacterium occur elsewhere?

Yes, *Lactobacillus crispatus* is also found in your gut and scientists have also found it in chickens. If you enter this bacterium in a search engine on the internet, you will probably come across a number of probiotics. After all, a lot of scientific research has already been done into the health effects of this bacterium.

What is this bacterium doing in my vagina?

Lactobacillus crispatus is very often associated with a healthy vagina. This bacterium produces a lot of lactic acid and therefore ensures acidity in the vagina. In this way, this bacterium protects your vagina against infections or pathogenic bacteria and fungi. *Lactobacillus crispatus* also makes other molecules that act as natural antibiotics or protect against inflammation, but not all these molecules are well known. When researching a healthy vaginal microbiome, we often focus on lactic acid, but each strain of *Lactobacillus* also produces an array of protective or beneficial molecules for our health.

Unravelling these molecules is something that Isala's team is happy to work on in the future. For example, we already know that *Lactobacillus crispatus* has a very good and active immune system so that this bacterium can protect itself against bacteriophages. These are viruses that can make (healthy) bacteria sick.

Supplementary Figure 5 - Information in layman's terms received by the participants. A webpage was dedicated to each of the top eight taxa. Here, the page on *Lactobacillus crispatus* is shown as an example. Other taxa can be accessed via https://isala.be/en/category/vaginal-bacteria/.

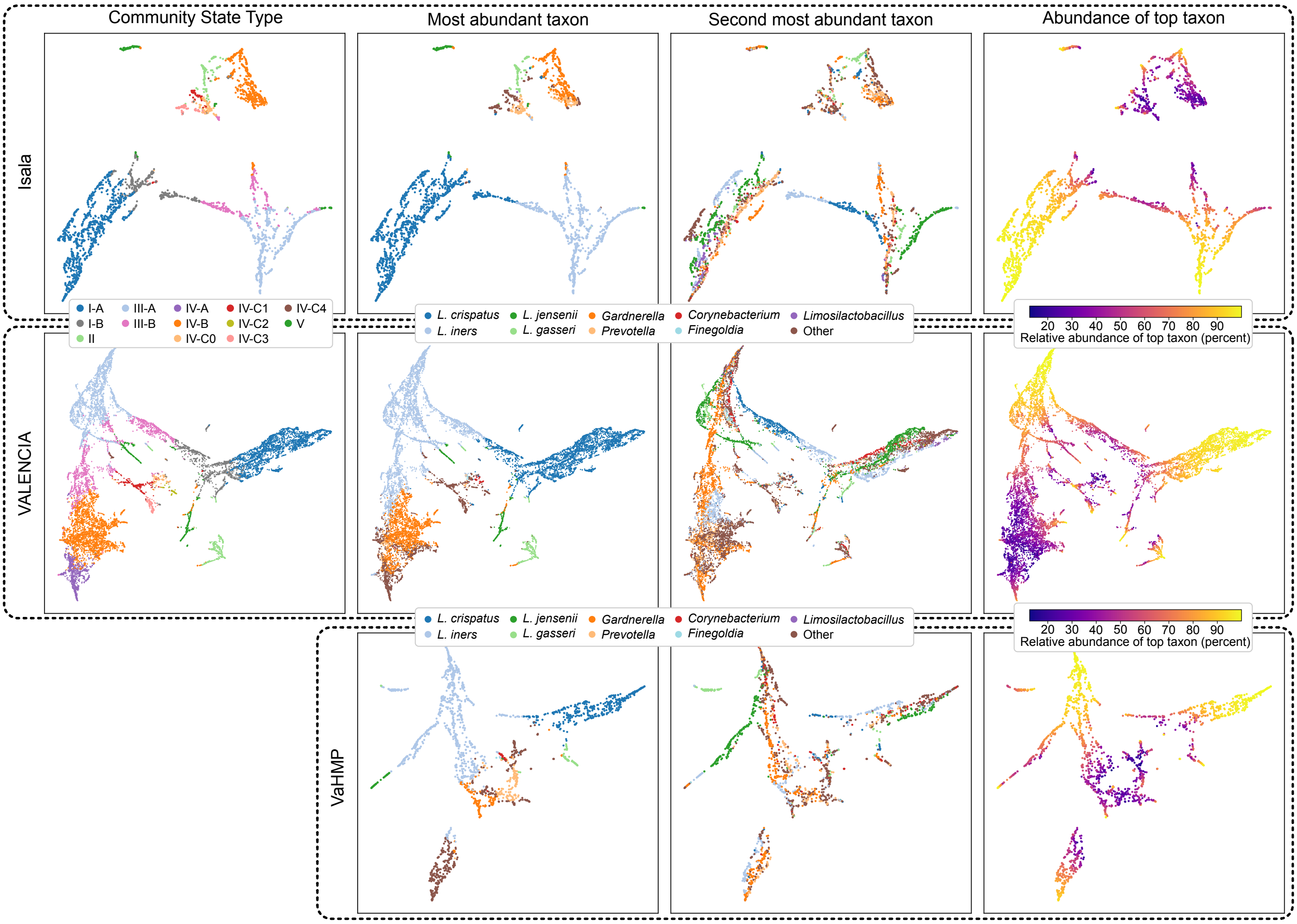

Supplementary Figure 6 – UMAP embedding of the Isala, VALENCIA and VaHMP datasets. As in Figure 3 of the main text, except that samples are now embedded with UMAP instead of t-SNE. Each point represents a sample, colored by a) community state type, b) most abundant taxon, c) second most abundant taxon or d) the relative abundance of the most abundant taxon.

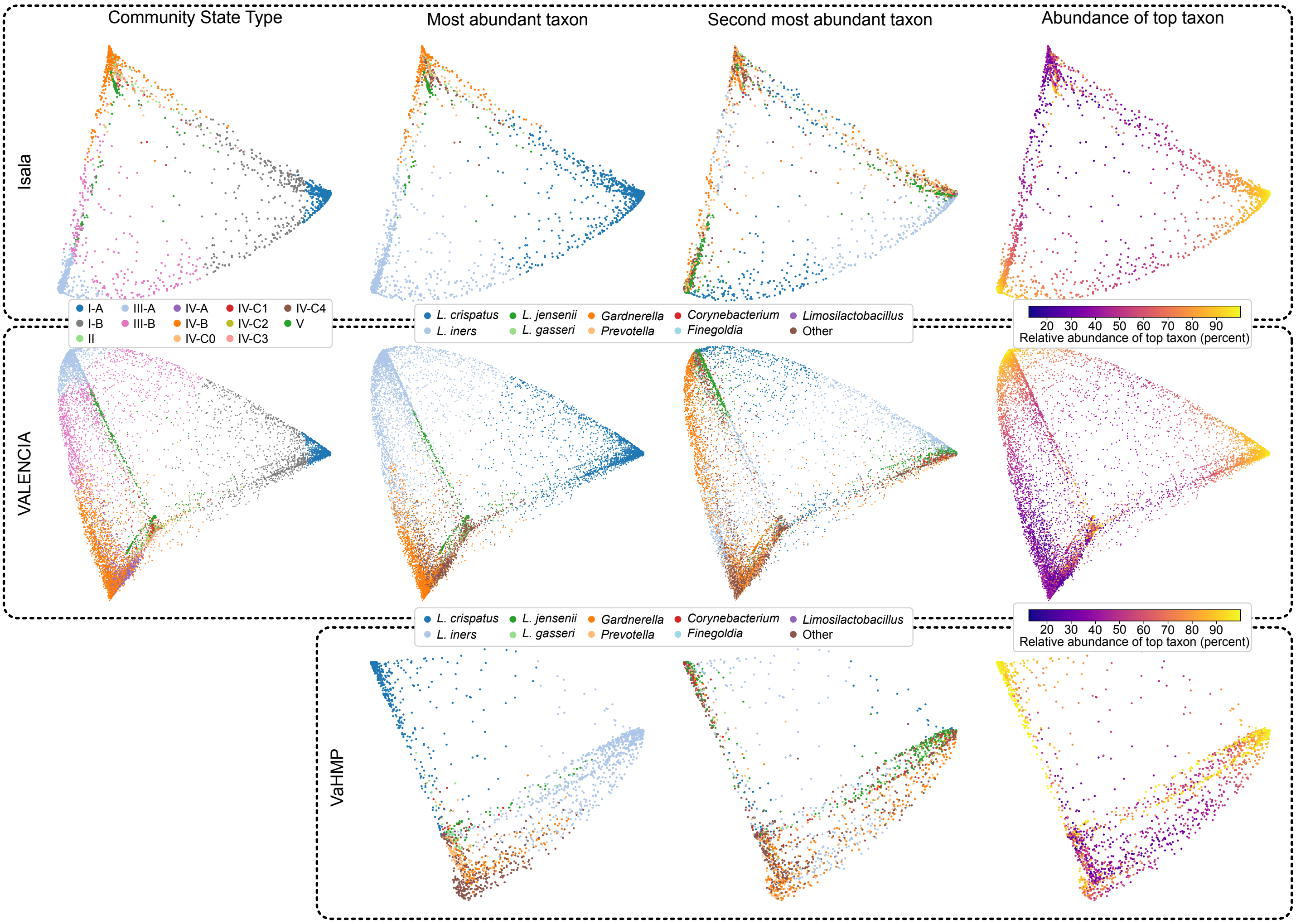

Supplementary Figure 7 – PCoA embedding of the Isala, VALENCIA and VaHMP datasets. As in Figure 3 of main text, except that samples are now embedded with PCoA instead of t-SNE. Each point represents a sample, colored by a) community state type, b) most abundant taxon, c) second most abundant taxon or d) the relative abundance of the most abundant taxon.

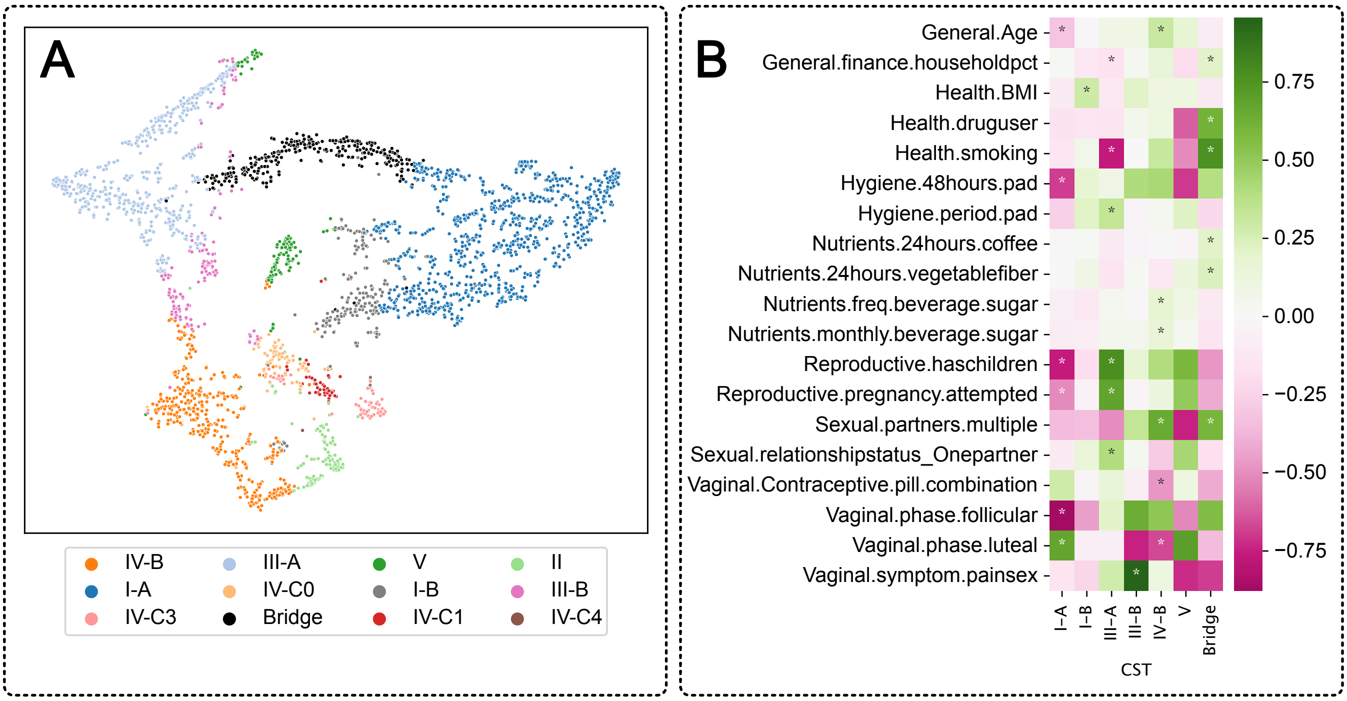

Supplementary Figure 8 – Analysis of the subset of samples in the bridge between CST I and CST III. A) The spatial distribution of these samples, indicated by black points. B) Association of these annotations against meta-data. An asterisk indicates a significant result (Linear model, p<0.05), corrected for multiple testing.

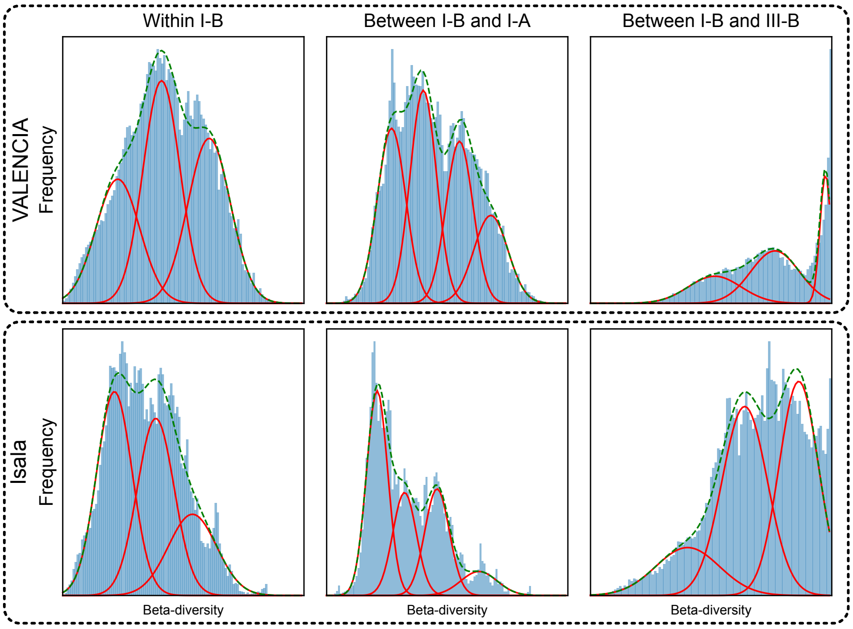

Supplementary Figure 9 - Distributions of beta-diversities within and between Community State Types in the VALENCIA dataset and the Isala dataset. Each plot is a histogram of Bray-Curtis distances between samples on (sub)genus level. Distances are shown between samples within CST I-B (column 1), between CST I-B and CST I-A (column 2) and between CST I-B and CST III-B (column 3). Within the I-B CST, a multi-modal distribution of beta-diversities indicates that there is a deeper sub-structure to the CST, implying that the CST may be further divided. Likewise, the boundary between the I-A and I-B CSTs appears as though there may be some samples which may need to be grouped into different CSTs, and likewise between CST I-B and III-B. Together, this indicates that: (1) CSTs may be further sub-divided; (2) CSTs may be partially merged; and (3) merged-CSTs may be differently sub-divided.

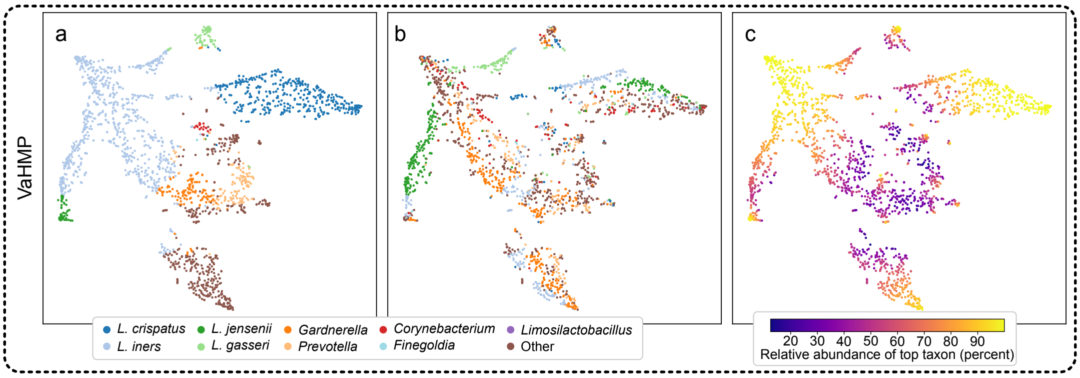

Supplementary Figure 10 – t-SNE embedding of the VaHMP dataset. t-SNE plot of all microbiome samples of the VaHMP dataset (multi-temporal samples per participant included), colored by (a) the most dominant genus, (b) the second most dominant genus, (c) and by the largest relative abundance level in each sample.

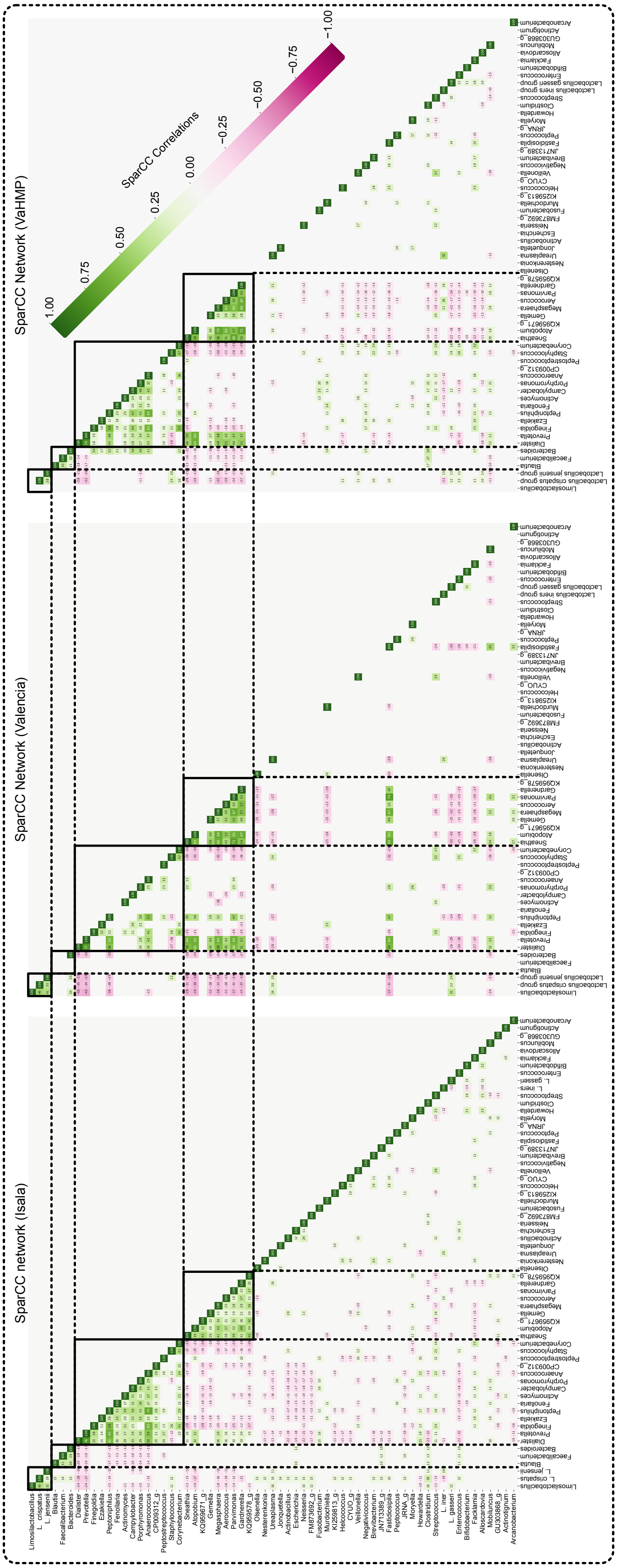

Supplementary Figure 11 – Aligned taxon correlation networks from SparCC. Correlations identified in Isala (left) are aligned with correlations found in VALENCIA (center) and VaHMP (right). Each identified module is highlighted with a black box. From top to bottom: *L. crispatus* module, *Bacteroides* module, *Prevotella* module, *Gardnerella* module. Each cell indicates the correlation (*100) for a pair of taxa. In VALENCIA and VaHMP, empty rows and columns indicate taxa that were not identified in the respective dataset (or were present in fewer than 10% of all participants).

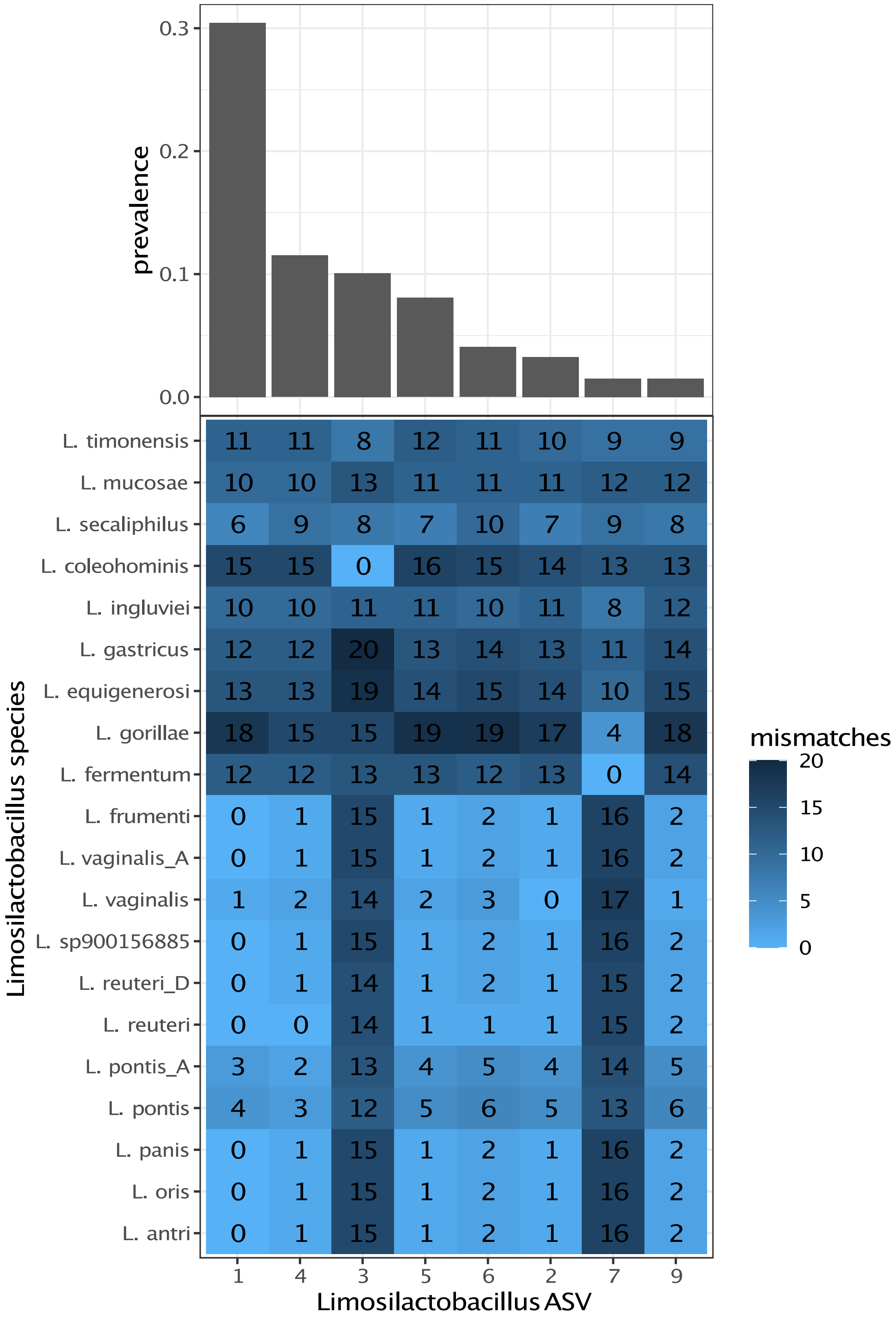

Supplementary Figure 12 – Subclassification of Limosilactobacillus ASVs. Columns represent *Limosilactobacillus* ASVs with a prevalence of at least 1%; rows represent *Limosilactobacillus* species in the 16S rRNA reference database of the GTDB (release 05-RS95) in order of their phylogeny. Each cell shows the number of nucleotide mismatches between a given ASV and the best matching 16S sequence of a given species. Only alignments of at least 250 nucleotides were considered. The bar chart shows the prevalence of each of the ASVs.

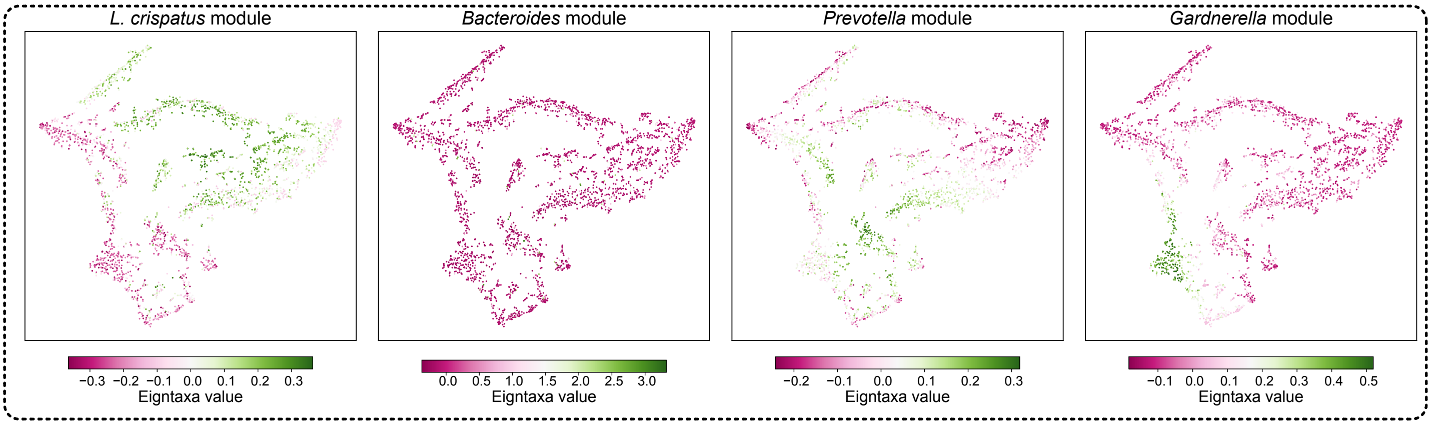

Supplementary Figure 13 – Eigentaxa values of the four identified co-occurrence modules projected onto the tSNE embedding. While the samples with high *L. crispatus*, *Prevotella* and *Gardnerella* module eigentaxa values are largely spatially defined, the samples with high *Bacteroides* module eigentaxa values are scattered across the tSNE embedding space.

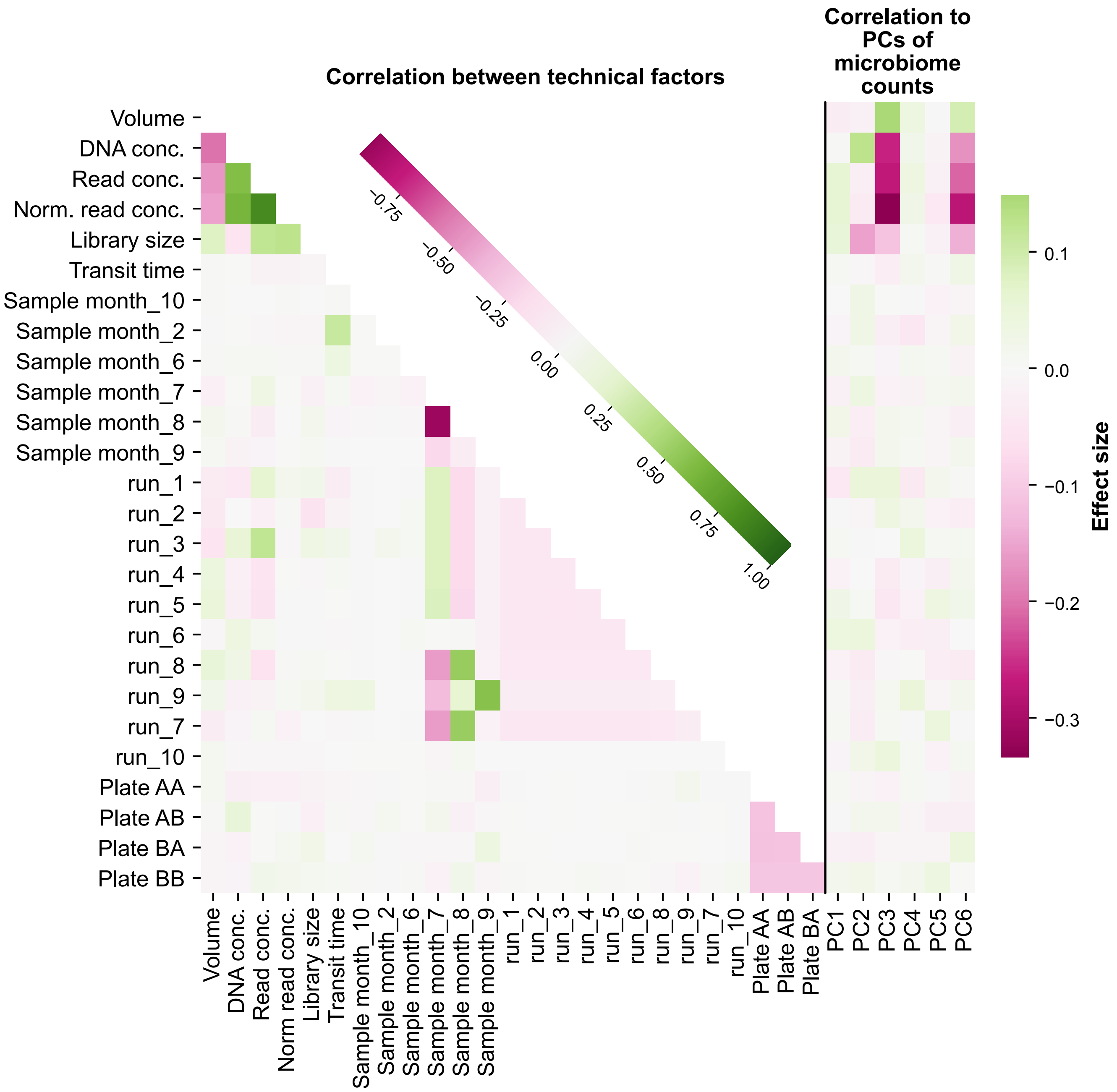

Supplementary Figure 14 – Associations between technical covariates and principal components of microbiome abundance data. Each row represents a technical covariate. The left section represents Pearson correlations between technical covariates. Asterisks indicate significant correlations (there are none). The right section indicates correlations between the first six principal components of a PCoA embedding of the microbiome data. The largest effects are found for Library Size, DNA concentration and read concentration, though none are significant.

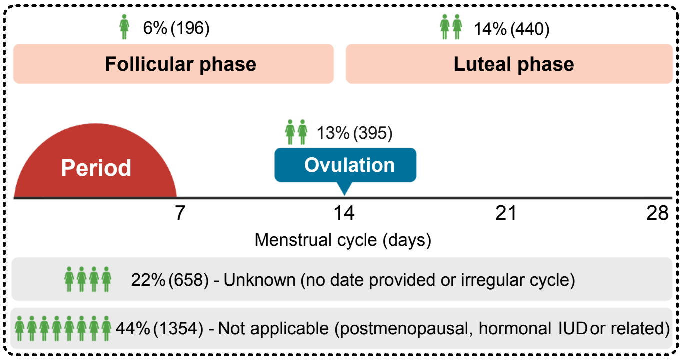

Supplementary Figure 15 – The menstrual cycle. Using information about each participant's cycle length and last menstruation, we estimated the stage of the cycle in which the swab was sampled to predict the levels of endogenous and exogenous levels of estrogen and progestin. Participants whose cycles had irregular lengths, or who did not report their last menstruation, were classified as "Unknown", and participants using hormonal contraceptives or were peri/post-menopausal were classified as "Not applicable".

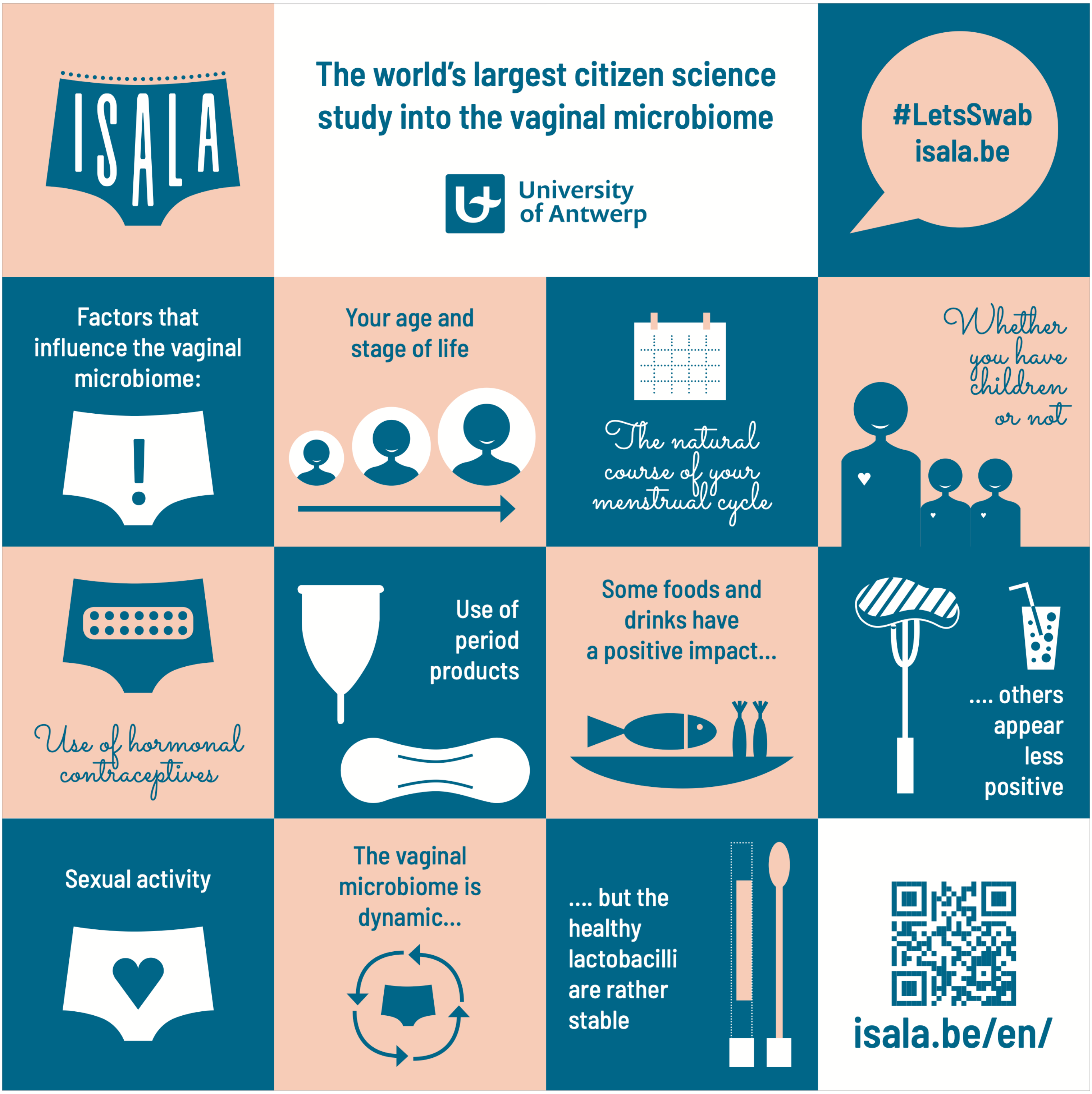

Supplementary Figure 16 – Example of an infographic communication on the possible associations with lifestyle factors. It is important to highlight that the infographic was accompanied by an explanation on the website to highlight not to overinterpret these associations (https://isala.be/en/results/#factors).

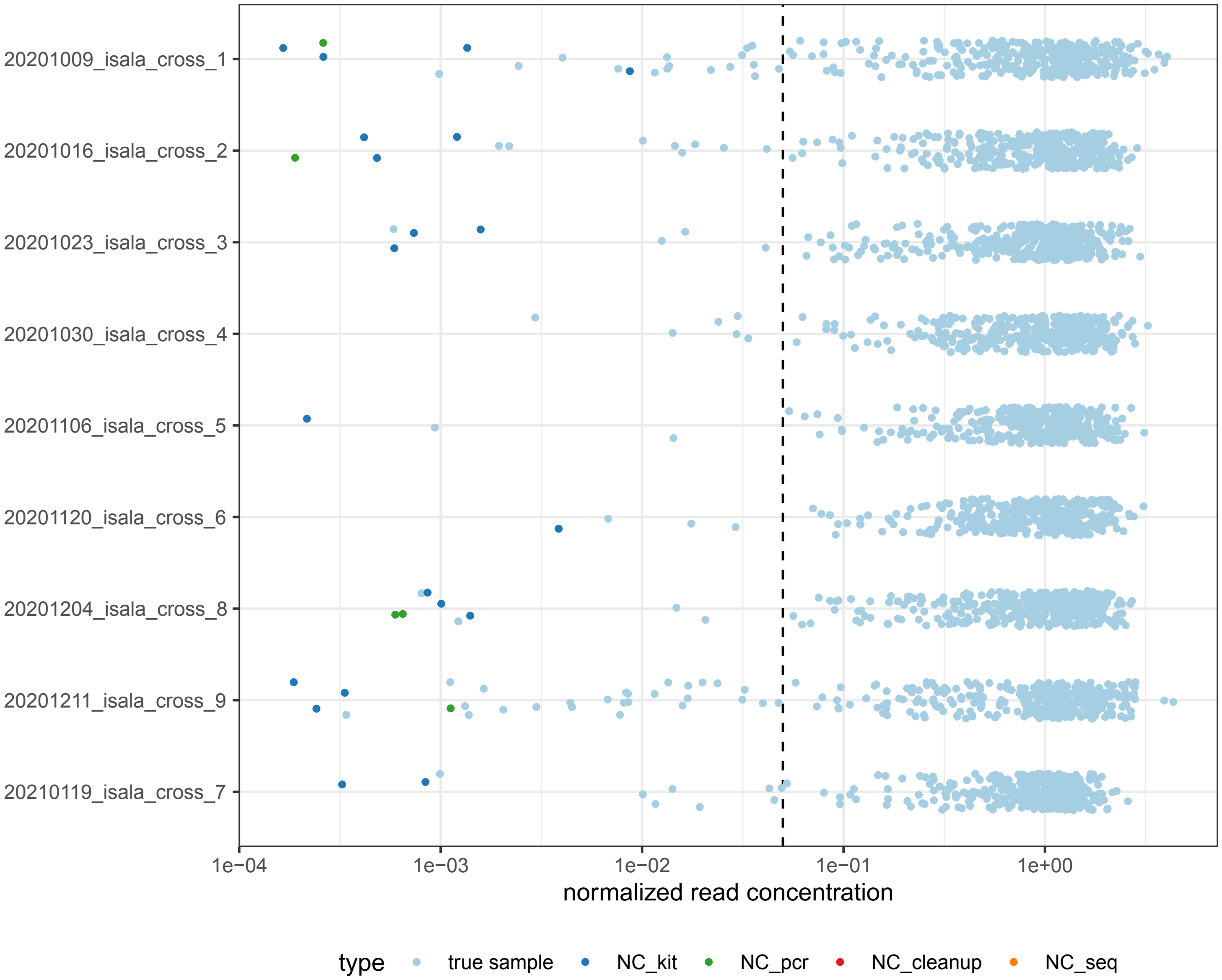

Supplementary Figure 17 – Quality control of the samples based on negative controls. Sequencing reads per volume of sample that was added to the sequencing library were calculated and normalized per sequencing run. The dashed line represents the cutoff of 0.05 that was used to select high-quality samples. NC_kit = DNA extraction controls, NC_pcr = PCR controls, NC_cleanup = PCR cleanup controls and NC_seq = sequencing controls (index reads that were unused on their respective run, but yielded some reads due to barcode leakage).

Supplementary Text 1

This survey was set up in collaboration with input from experts (mainly from team Prof. Lebeer for microbiome-related parameters, Prof. De Backer for communication- and food-related questions, Dr. Masquiller for sociology-related questions, Sensoa for sexual-lifestyle related questions, Provincial Institute for Hygiene for general health-related questions, Prof. Karen Scott for food-related questions) and non-experts (citizens) in the context of Flanders in the year 2020. Questions were asked in a standardized way when possible via the Qualtrics platform, but always taking the 'citizen-science aspect' of the project into account. The study was not set-up as a population study, but as a citizen-science project. It reflects a specific geography and zeitgeist.

Translated from the original Dutch survey Version 4 from the 14th of April 2020

Integrated genetic and functional analysis of the female microbiome in a Flemish cohort

Research study at the University Hospital Antwerp and the University of Antwerp, in a collaboration between the Faculty of Sciences, Department of Bioengineering and the Faculty of Medicine and Health Sciences

It takes about 40 minutes to complete the survey. All questions in the survey probe factors that influence bacteria on your body and are important to correctly interpret Isala's results. Therefore, we ask you to complete all the questions. The survey is designed so that your personal data cannot be directly linked to your answers to ensure your privacy. If you find it difficult to complete this survey, you can always contact us at isala@uantwerpen.be

General

Your personal study number: ....

Gender:
- o Female
- o Male
- o Transman: since when?...(year)
- o Transwoman: since when?.... (year)
- o Other

Age: ....years

Height: .... cm

Weight: ....kg

This is how best to describe my current relationship status:
- o Permanent partner
- o Permanent partner, not living together
- o Alternating partners
- o Single

My partner(s) is/are...

- Man
- Woman
- Both
- Neither

This is what my family looks like:

- Living together with partner or married
    - If yes, how long have you lived together (please circle the appropriate answer)
        - Less than 3 months
        - Between 3 months and 1 year
        - Between 1 year and 5 years
        - Between 5 and 10 years
        - More than 10 years
- Living together with friends or other housemates
    - If yes, how long have you lived together (please circle the appropriate answer)?
        - Less than 3 months
        - Between 3 months and 1 year
        - Between 1 year and 5 years
        - Between 5 and 10 years
        - More than 10 years
- Living with relatives
    - If yes, how long have you lived together (please circle the appropriate answer)?
        - Less than 3 months
        - Between 3 months and 1 year
        - Between 1 year and 5 years
        - Between 5 and 10 years
        - More than 10 years
- Single parent
- Single parent with co-parenting
- Living alone

If you are not living alone, how many people do you live with in the same house, not including yourself (even if not all the time)?.......................

Do you have any children?

- Biological children: how many?............................
    - How did you give birth?
        - By caesarean section:..... times
        - Natural childbirth:...... times
- Adopted children or foster children: how many?............................
- Plus children (= stepchildren) : how many?...........................
- No children

How would you describe the area where you grew up? This question is about where you spent most of your childhood. If in doubt, choose the place where you first spent a long time.

- Town centre
- Village centre
- Residential area
- Busy lane
- Rural area
- Green zone/recreational area/....
- Industrial zone

How would you describe the area where you live <u>now?</u>

- Town centre
- Village centre
- Residential area
- Busy lane
- Rural area
- Green zone/recreational area/....
- Industrial zone

Do you come into an environment every day with....

- A lot of greenery
- A lot of greenery
- Some greenery
- Little green
- Very little greenery
- No green

Were you born in Belgium?

- Yes
- No
  - Which country were you born in?..........................
  - How old were you when you came to Belgium?...........................
  - Do you live permanently (more than 6 months) in Belgium? ....

In which country(ies) did you live until the age of three? ....

What nationality(ies) does your birth mother have? ....

What nationality(ies) does your biological father have?...................

Are there other cultures/countries you also identify with besides Belgium?

- Yes, which ones?..........................
- No

Were you abroad (for at least three consecutive days) in the past three months?

- Yes, to which country/countries? ....
- No

What is the highest degree you obtained?

- Primary school

- ○ Secondary education: ASO
- ○ Secondary education: KSO
- ○ Secondary education: TSO
- ○ Secondary education: BSO
- ○ University: bachelor
- ○ University: bachelor/master (candidate/licensee)
- ○ Postgraduate: doctor
- ○ Other: ....
- ○ No

In which sector do you work?

- ○ Hospitality and tourism
- ○ Education
- ○ Event agencies, audiovisual and film sector
- ○ Social economy
- ○ Consultancy and other services to companies
- ○ Real estate and facility services
- ○ Food, tobacco
- ○ Transport, logistics
- ○ Textiles, clothing
- ○ Industry (general)
- ○ Government and public administration
- ○ Socio-cultural sector, sports, youth work, ...
- ○ Agriculture, horticulture, forestry, fishery
- ○ IT services and companies
- ○ HR and employment agencies
- ○ Wood and furniture industry
- ○ Graphic, paper and cardboard industry
- ○ Health services and institutions
- ○ Energy, water
- ○ Chemistry, metal
- ○ Construction
- ○ Banking, insurance, postal services, telecoms
- ○ Trade, rental
- ○ Other: ....

Describe your work situation under normal circumstances:

	**Daily**	**More than 3x/week**	**Weekly**	**Monthly**	**Rarely**	**Never**
Contact with other people	○	○	○	○	○	○
Contact with chemicals (e.g., chlorine, flame retardants, Teflon, acetone, disinfectant, lab material, ...)	○	○	○	○	○	○

Physical contact with outdoor plants	○	○	○	○	○	○
Physical contact with indoor plants	○	○	○	○	○	○

Do you have contact with animals while at work or at home?

- ○ Yes,
  - At work, how many and which animals?..
  - I have pets,
  - Indoors, how many and which animals?..
  - Outdoors, how many and which animals?..
- ○ No

Please indicate your work rhythm. If you combine several jobs, add everything up (multiple answers are possible)

- ○ Full-time
- ○ Part-time: ....%
- ○ Long-term sick leave (more than three months)
- ○ Unemployed
- ○ (Early) retirement
- ○ Student
- ○ Voluntary work
- ○ Other: ....

I usually work (several answers possible)

- ○ During the day
- ○ at night
- ○ In shifts:
  - Early shift
  - Late shift
  - Nights
- ○ Other: ....

Do you smoke?

- Yes, approximately how many cigarettes do you smoke per day?.................................
  - ○ How long have you been smoking?...........................(years/months)
- No, ex-smoker
  - ○ Approximately how many cigarettes did you smoke per day before? ....
  - ○ How many years/months ago was this? ....
  - ○ How long did you smoke?....................(years/months)
- No, never smoked

Do you use drugs (excluding alcohol)? Please also indicate which ones. This information is processed pseudonymously. We have the utmost respect for you and for your privacy.

- Yes, which drugs?.................................
  - ○ What is the frequency of your current drug use?
    - ▪ Several times a day
    - ▪ Daily

        - More than 3x/week
        - Weekly
        - Monthly
        - Rarely
    - How long have you been using drugs? ....(years/months)
- No, ex-drug user: what drugs?................
    - What was the frequency of your drug use at the time?
        - Several times a day
        - Daily
        - More than 3x/week
        - Weekly
        - Monthly
        - Rarely
    - How many years/months ago was this? ....
    - How long have you been using drugs? ....(years/months)
- No, never used drugs

On average, how many hours do you sleep per night?
- On weekdays:..........h.
- On days off: ....h.

How many hours a week do you exercise (multiple answers possible)?
- I do no sport
- Moderate physical activity: ....h.
- Intense physical activity: ....h

Which sports do you practice? ....

On average, how many minutes/hours a day do you spend outdoors?
- During the week.............................
- On weekends.................

Diet

Do you take probiotics or "good bacteria"? (both food and drink) (multiple answers possible)
- In dairy products such as Yakult, Actimel?
    - Yes
        - How often? several times a day/ daily/ more than 3 times a week/ weekly/ monthly/ rarely/ never
        - Which products? ....
        - Have you used such products in the last month? ....
    - No
- In capsules such as Enterol, Probactiol or other similar dietary supplements from pharmacies or drugstores?
    - Yes

        - How often? several times a day/ daily/ more than 3 times a week/ weekly/ monthly/ rarely/ never
        - Which products? ....
        - Have you used such products in the last month? ....

- In yoghurt?
    - Yes
        - How often? several times a day/ daily/ more than 3 times a week/ weekly/ monthly/ rarely/ never
        - Which products? ....
        - Have you used such products in the last month? ....
    - No

How regularly did you eat or drink the items below in the past 3 months?

	**Multiple times a day**	**Daily**	**More than 3x/week**	**Weekly**	**Monthly**	**Rarely**	**Never**
Dairy products (cheese, milk, yoghurt, buttermilk, etc.)	○	○	○	○	○	○	○
Fermented foods (olives, salami, sauerkraut, sourdough bread, pickles, ...)	○	○	○	○	○	○	○
Alcohol	○	○	○	○	○	○	○
Meat	○	○	○	○	○	○	○
Animal products (eggs, cheese, gelatine,...)	○	○	○	○	○	○	○
Fish	○	○	○	○	○	○	○
Sweet drinks with sugar (e.g. soft drinks, fruit juice)	○	○	○	○	○	○	○
Sugar-free sweet drinks (e.g. light soft drinks)	○	○	○	○	○	○	○
Fruits	○	○	○	○	○	○	○
Vegetables	○	○	○	○	○	○	○

When you drink alcohol, what do you usually drink? (multiple answers possible)

- Wine and/or sparkling wine
- Beer
- Strong drinks
- I do not drink alcohol
- Other:................

Do you take any vitamins, minerals or other supplements? If yes, please also fill in which ones?

- Yes regularly throughout the year: which ones? ....
- Yes sometimes, during a certain period: which ones?...............
- No

General health

How were you born?

- By caesarean section
- With a vaginal delivery
- Do not know

What is your blood type? (Circle your rhesus factor if you know)

- A : + / -
- B: + / -
- AB : + / -
- O : + / -
- Do not know

How do you rate your general state of health?

- Very good
- Good
- Fair
- Bad
- Very bad

Do you have any of the following conditions? (Please state the condition)

**System/all over your body**<br>e.g., rheumatism, arthritis, MS, ...	
**Skin**<br>e.g., Psoriasis, acne,…	
**Heart/Blood vessels**<br>e.g., endocarditis, aneurysm, …	
**Stomach/Intestines**<br>e.g., Crohn's disease, colitis, ...	
**Airways**<br>e.g., asthma, allergies, …	
**Eyes**<br>e.g., Glaucoma, cataracts, ...	
**Nose-Throat-Ear**<br>**e.g., chronical sinusitis**	
**Hormonale (eg. thyroid) or diabetes (type 1/type 2)**	
**Reproductive system**<br>e.g., Endometriosis	
**Blood**<br>e.g., Anaemia, …	
**Cancer**<br>e.g., Leukemia, breastcancer, …	Subquestion: How many years/months ago was your diagnosis?...........
**Others**	

Have you ever received any of the following diagnoses?

- Allergy
  - Asthma
    - In the last 12 months, how many episodes of wheezing did you have that were not related to a cold?
      - None
      - 1-3
      - 4-12
      - More than 12
  - Hay fever (pollen allergy)
  - House dust mite allergy
  - Animal allergy, which one? ....
  - Other inhalation allergy: ....
    - How many episodes of runny nose and/or itchy eyes did you have in the last 12 months, related to an inhalation allergy (pollen, house dust mite, animals, ...)?
      - Less than 4 days/week or less than 4 weeks/year, and NOT interfering with daily activities
      - Less than 4 days/week or less than 4 weeks/year, and interfering with daily activities
      - More than 4 days/week and more than 4 weeks/year, and NOT disruptive to daily activities More than 4 days/week and more than 4 weeks/year, and interfering with daily activities
      - No
  - Eczema
    - How often do you have flare-ups of eczema?
    - Several times a day
    - Daily
    - Weekly
    - Monthly
    - Rarely
  - Food allergy to ....
  - Other: ....
- Eating disorder
  - Anorexia nervosa
  - Bulimia nervosa
  - Binge Eating Disorder
  - Other: ....
- Other condition: ....
- No

Are you lactose intolerant?

- Yes
- No

Do you have a gluten allergy?

- Yes
- No

Have you had any antibiotic or antimycotic2 treatment in the past three months (also report vaginal antibiotics and antimycotics such as e.g. gynodaktarin please)?

- Yes, how many times?.................
- No

When did your last antibiotic treatment take place (approximately)?...

Did you take prescription medication in the last three months? If yes, can you indicate how many different products were involved?

Name	Dosis	Startdate	Stopdate	Reason

Have you been to the dentist in the last 12 months?

- Yes
- No

Have you suffered from regular bleeding gums in the last 12 months?

- Yes
- No

Have you suffered from caries or cavities in the last 12 months?

- Yes
- No

How did you feel in the past four weeks?

Question	Constantly	Mostly	Often	Sometimes	Rarely	Never
Did you feel very nervous?	○	○	○	○	○	○
Were you so badly down that nothing could cheer you up?	○	○	○	○	○	○
Did you feel calm and peaceful?	○	○	○	○	○	○
Did you feel dejected and gloomy?	○	○	○	○	○	○
Did you feel happy?	○	○	○	○	○	○
Did you feel stressed?	○	○	○	○	○	○

Reproductive and sexual health

In the past three months, have you had one or more sexual partner(s)?

- o Yes, one steady partner
- o Yes, several regular partners : how many?...................................
- o Yes, alternating partners : how many?................................
- o No

If yes, was this sexual contact with :

- o Woman(s)
- o Man(s)
- o Woman(s) and man(s)

What sexual acts have you performed in the past 3 months? (multiple answers possible)

- o Manual sex
- o Oral sex
- o Vaginal penetration with
  - o Fingers
  - o Toys
  - o Other: ....
- o Anal sex
- o Other: ....

What sexual acts have you received in the past 3 months? (multiple answers possible)

- o Manual sex
- o Oral sex
- o Vaginal penetration with
  - o Fingers
  - o Penis
  - o Toys
  - o Other: ....
- o Anal sex
- o Other: ....

Have you had vaginal sex in the past 3 months, and if so, how frequently?

- o None
- o One exceptional time
- o About once a month
- o Several times a month
- o About once a week
- o Several times a week
- o Daily

Do you have vaginal sex during your monthly bleeding?

- o Yes
- o No
- o Sometimes

Did you experience any problems fulfilling your pregnancy wish at that time?

- Yes
    - Did you experience any problems with this at the time?
    - No
    - Yes
        - If Yes, did you see your GP/gynaecologist for this?
            - Yes
            - No
- No

Has a fertility programme ever been initiated for you? (multiple answers are possible)

- Yes, because of my reduced fertility with known cause, i.e. ....
- Yes, because of my partner's reduced fertility
- Yes, because of unexplained reduced fertility
- Yes, because me and my female partner want(s) to become mothers
- Yes, because I want(d) to become a mother without a partner
- Yes, because of another reason ....
- No

If yes, what type(s) of fertility programme(s) did you follow? (multiple answers are possible)
Hormone therapy

- Artificial insemination
- In vitro fertilisation (IVF)
- Intracytoplasmic sperm injection (ICSI)
- Other:.......................................

How many times have you gone through such a process? ....

How many trajectories/cycles of these resulted in pregnancy? ....

How many trajectories/cycles of these resulted in the birth of a child? ....

How many times were you pregnant?...................................

Please provide more explanation for each pregnancy in the table below

1[e]	Was the pregnancy planned?<br>o Planned<br>o Unplanned<br>Did you carry the pregnancy to term (until at least 37 weeks' gestation)?<br>o Yes<br>o No, premature birth at ... weeks<br>o No Aborted, spontaneous miscarriage at .... weeks<br>o No Aborted, miscarriage with medication at .... weeks<br>o No aborted, curettage miscarriage at ... weeks<br>o No aborted, abortion at .... weeks
2[e]	Was the pregnancy planned?<br>o Planned<br>o Unplanned<br>Did you carry the pregnancy to term (until at least 37 weeks' gestation)?

	o Yes<br>o No, premature birth at ... weeks<br>o No Aborted, spontaneous miscarriage at .... weeks<br>o No Aborted, miscarriage with medication at .... weeks<br>o No aborted, curettage miscarriage at ... weeks<br>o No aborted, abortion at .... weeks
3e	Was the pregnancy planned?<br>o Planned<br>o Unplanned<br>Did you carry the pregnancy to term (until at least 37 weeks' gestation)?<br>o Yes<br>o No, premature birth at ... weeks<br>o No Aborted, spontaneous miscarriage at .... weeks<br>o No Aborted, miscarriage with medication at .... weeks<br>o No aborted, curettage miscarriage at ... weeks<br>o No aborted, abortion at .... weeks
4e	Was the pregnancy planned?<br>o Planned<br>o Unplanned<br>Did you carry the pregnancy to term (until at least 37 weeks' gestation)?<br>o Yes<br>o No, premature birth at ... weeks<br>o No Aborted, spontaneous miscarriage at .... weeks<br>o No Aborted, miscarriage with medication at .... weeks<br>o No aborted, curettage miscarriage at ... weeks<br>o No aborted, abortion at .... weeks
…	

Did you have a microbial infection during one of your pregnancies?

- Yes
  - If yes, which one?
    - Cytomegalovirus (CMV)
    - Toxoplasmosis
    - Other:...........
- No
- I do not know

Are you currently breastfeeding?

- Yes
- No

What remedies have you used in the last three months to avoid getting pregnant? Please also indicate which brand you used (multiple answers possible)

- Contraceptive pill:........................
- Mini pill:........................
- Hypodermic pill:........................
- Contraceptive patch (e.g. Evra) :........................

- o Contraceptive ring (e.g. Nuvaring) :.........................
- o Hormonal implant (e.g. Implanon) :.........................
- o Hormonal coil (e.g. Mirena): ....
- o Copper IUD:.........................
- o Condom:.........................
- o Pessary (= rubber dome) :.........................
- o Periodic abstinence (= not having sex in fertile period)
- o Coitus interruptus (= withdrawing penis before ejaculation)
- o Other:.........................
- o No

Are you currently using any other method?
- o No
- o Yes, which one?............................. (same answer options as above)

Why haven't you used a contraceptive method in the last three months?
- o I did not have sex
- o I did not have sex from which I could get pregnant
- o I am infertile or reduced fertility
- o I would like to get pregnant
- o Contraception is expensive
- o My partner is infertile or less fertile
- o My partner uses contraception
- o My partner has been sterilised
- o I have been sterilised
- o I don't like to use it
- o My partner does not want to use contraception
- o Using contraception is not the norm within my culture
- o I do not use contraception for religious reasons
- o I have a female partner
- o Others:...................

First day of your last period (= first day of monthly bleeding): ..../........./...........

Do you currently have your monthly bleeding at regular intervals?
- o Yes
- o No
    - o Is this related to any of the following possibilities?
        - I have no/never monthly bleeding
        - I have yet to have monthly bleeding after giving birth
        - I am in my transition period (period just before menopause)
        - I am in my menopause
        - I am continuing my hormonal contraception
        - I don't know
        - Other: ....

On average, how many days are there between the first day of your period and the first day of your next one?

- I do not use hormonal contraception or any other hormonal treatment that may affect my menstrual cycle: ....
- I use hormonal contraception or another hormonal treatment that may affect my menstrual cycle
- I do not have a menstrual cycle
- I do not know

Do you have pain (e.g. headache, backache, lower abdominal pain, ...) during monthly bleeding? If yes, also describe what pain you have.

- No, I never have pain
- Yes, I always have pain
  - Do you use painkillers to deal with the pain?
    - No
    - Yes, which and how many a day of painkillers ?........................
- Yes, I have pain sometimes
  - Do you use painkillers to deal with the pain?
    - No
    - Yes, which and how many a day of painkillers ?........................

Do you experience pain on the days around your ovulation (about 14 days after the first day of your period)?

- Yes
- No
- Sometimes
- Do not know

During my monthly bleeding, I use (multiple answers possible):

- Tampon
- Panties
- Menstrual cup
- Panty liner
- Other: ....

In the last month, have you experienced one or more complaints on your vulva or vagina (multiple answers possible)?

- No
- Redness
- Swelling
- Pain (general)
- Itching
- Burning sensation
- Increase in vaginal discharge (different from normal monthly discharge)
- Change in vaginal discharge (different colour, unpleasant smell)

- o Pain during sexual contact
- o Other:..................................

If an answer indicated, please indicate how severe

- o No
- o Mild complaints
- o Moderate complaints
- o Severe complaints

Have you ever suffered from one or more of the following conditions? (multiple answers possible)

Condition	No	I don't know	Yes, now	Yes, once
PCOS (polycycsteus ovarium syndrome)	○	○	○	○
Endometriosis	○	○	○	○
Chlamydia	○	○	○	○
Gonorroe	○	○	○	○
Bacterial vaginosis	○	○	○	○
Aerobic vaginitis	○	○	○	○
Herpes	○	○	○	○
Mycoplasma	○	○	○	○
Fungal infection	○	○	○	○
Genital warts	○	○	○	○
Trichomonas vaginalis	○	○	○	○
Bladder infection	○	○	○	○
Other:……	○	○	○	○

If yes,

Were you treated for this? (multiple answers are possible- please indicate for which condition)

- o Yes, by means of antibiotics, for ....
- o Yes, by means of antimycotics (product against fungal infections), for ....
- o Yes, by means of probiotics, for ....
- o Yes, by means of another product: .... For ....
- o I do not know
- o No

To your knowledge, have your mother/sister(s)/daughter(s)/other female relatives, already had a similar condition?

- o Yes, what is your relationship?.............................
- o No
- o Don't know

In the last three months, have you had a change in your lifestyle (e.g., moving house, new partner, change in diet, change in exercise, stressful periods, etc.)?

- Yes, specifically ....
- No
- Do not know

Which of the following products have you ever used? (multiple answers possible)

- Vaginal antimycotic (e.g. Gynodaktarin, Clotrimazole, Canestene, ...)
  - How often? Several times a day / daily / weekly / monthly / rarely / no more
  - Do you still use this product?
    - Yes
    - No
- Vaginal soap (e.g. Lactacyd femina, Multi-Gyn, Sebamed, ...)
  - How often? Several times a day / daily / weekly / monthly / rarely / no more
  - Do you still use this product?
    - Yes
    - No
- Vaginal probiotics (e.g. Physioflor, Pro-Ven, Mucogyne, Gynoflor, ...)
  - How often? Several times a day / daily / weekly / monthly / rarely / no more
  - Do you still use this product?
    - Yes
    - No
- None of the above

Have you ever had a vaginal smear taken by your doctor or gynaecologist?

- Yes
- No
- Do not know

Have you been vaccinated against HPV (human papillomavirus)?

- Yes
- No
- Do not know

In some cultural/religious communities, girls and women are circumcised. Does this happen in your community?

- Yes
- No
- Do not know

Have you undergone female circumcision?

- Yes
- No

Have you ever had surgery on your vagina or vulva? We do not mean the uterus or ovaries here.

- Yes
- No

Have any operations been performed on your female genital organs for non-medical reasons (e.g. pricking, piercing, tattooing, etc.)?

- Yes
- No

Have you ever been concerned about your vaginal health?

- Yes
- No
- Somewhat

With whom do you feel you can talk openly about your vaginal health? (multiple answers possible)

- Partner
- Friends/friends
- Children
- Colleagues
- GP
- Gynaecologist
- Other: ...
- Nobody

Personal hygiene

Thinking about the past three months, can you indicate how often you did the actions below?

Question	Daily	More than 3x/week	Weekly	Monthly	Rarely	Never
How often do you shower	○	○	○	○	○	○
How often do you use body-lotion/milk?	○	○	○	○	○	○
How often do you use handcreme?	○	○	○	○	○	○
How often do you use face creme?	○	○	○	○	○	○
How often do you use peelings or scrubs for your skin (face/body)?	○	○	○	○	○	○
How often do you use antibacterial soap or desinfectants for your hands?	○	○	○	○	○	○
How often do you use medicinal cremes (eg.	○	○	○	○	○	○

Cortisone or antibiotic cremes)?						
How often do you use sunscreen?	○	○	○	○	○	○
How often do you use parfume?	○	○	○	○	○	○
How often do you use deodorants?	○	○	○	○	○	○
How often do you use shaving soap or other shaving products?	○	○	○	○	○	○

Do you have sensitive skin?

- Yes
- No

What skin products do you normally use on a daily basis? (multiple answers possible)

- Cosmetics
- Non-medicated creams (e.g. day cream, body lotion, ...)
- Medicated creams
- Soaps
- Deodorants
- Perfume
- Scrub
- Other:....................
- No

Do you suffer from excessive sweating?

- Yes
- No

Does it coincide with specific period in your menstrual cycle?

- Yes
- No
- Do not know

How often do you shave your pubic area?

- Daily
- More than 3x/week
- Weekly
- Monthly
- Rarely
- Never

During the last three months, have you performed vaginal flushing (by injecting a liquid into your vagina - also known as vaginal showering)?

- Yes
- No

Studies have shown that certain ties (kinship, cohabitation, etc.) between individuals can have an impact on the microbiome. Therefore, we would also like to investigate this with Isala. Do you agree that your results may be linked to someone in your family or with whom you live who is also participating in Isala? Provided you have both given permission. You will not be able to see each other's answers and results.

- I agree
- I do not agree

Does anyone in your extended family or with whom you live participate in Isala? If yes, please also indicate the number of people involved.

- Yes: how many? ...
- No

(If yes) Please indicate the name and relationship with this person in the table below.

		Connected in this way:
Surname	Name	

We asked a lot about your lifestyle factors and your habits over the past 3 months but we know that the coronavirus had a big effect on many lives and habits. Anything you want to say about it?

....

....

....

Do you have any comments for us after completing this survey?

....

....

....

Confidentiality

The protection of personal data is determined by law by the Privacy Commission. All information is kept confidential in accordance with the Privacy Act. Participants will be identified by code. If the results of this study are published in a report or scientific journal, no name will be mentioned.

**Short follow-up SURVEY at time of sampling (June 2020)
Integrated genetic and functional analysis of the female microbiome in a Flemish cohort**

Research study at the University of Antwerp and Antwerp University Hospital, in a collaboration between the Faculty of Sciences, Department of Bioengineering and the Faculty of Medicine and Health Sciences

Completing this additional survey will take about 5 minutes. Indeed, due to the Corona crisis and its impact on our lifestyle, it proved necessary to ask some additional questions or to take a quick poll to see if things might have changed. The survey is still designed so that your personal data cannot be directly linked to your answers to ensure your privacy. Be sure to let us know if you have any additional questions when completing the survey by contacting us at isala@uantwerpen.be.

General
Weight: ....kg

Has your sexual relationship status changed recently?
- No, I have the same sexual partner
- Yes, I have another sexual partner
- Yes, I have multiple sexual partners
- Yes, I no longer have a sexual partner

Can you make ends meet monthly with the sum of total disposable family income (all inclusive)?
- Very difficult
- Difficult
- Rather difficult
- Rather easy
- Easy
- Very easy

In 2020, what was the total household disposable income (all inclusive2) per month (in a normal month - outside the COVID-19 period such as January or February 2020)?
- Amount approximately per month in euros:............
- I don't know
- I don't want to say

How many people depend on this income? ....

How big is your share of the family income?
- I am the only one contributing
- I have the largest share
- We contribute equally
- I contribute the least
- I do not contribute

Diet

Did you take probiotics or yoghurt with live ferments (e.g. Pur Natur) 24h before taking your vaginal swab?

- o Yes, which one? ....
- o No

Did you drink so much in the last 24h that you had to get up at night to pee?

- o Yes
- o No

How many servings did you eat or drink of the items below in the past 24h (serving = a handful or glass)?

	None	1 portion	2 portions	3 portions	More than 3 portions
Dairy products (cheese, milk, yoghurt, buttermilk, etc.)	○	○	○	○	○
Fermented foods based on lactic acid bacteria (olives, salami, sauerkraut, kimchi, pickles, ...)	○	○	○	○	○
Alcohol	○	○	○	○	○
Meat	○	○	○	○	○
Fish	○	○	○	○	○
Coffee					
Sweet drinks with sugar (e.g. soft drinks, fruit juice)	○	○	○	○	○
Sugar-free sweet drinks (e.g. light soft drinks)	○	○	○	○	○
Fruits high in fibre or possible prebiotics: bananas, kiwi, nectarine, peach, persimmon, raspberries, apples, pears, oranges, pomegranate, grapefruit, cranberries (also dried), prunes, sultanas	○	○	○	○	○
Vegetables high in fibre and possible prebiotics: onion, chicory, asparagus, garlic, leeks, peas, beans, artichokes, savoy cabbage, avocado	○	○	○	○	○
Cold pasta (source of resistant starch)	○	○	○	○	○
Whole-wheat bread	○	○	○	○	○

Sour dough bread					
Quinoa					
Chia seeds, linseed, pumpkin seeds, poppy seeds, sunflower seeds					
Nuts (almonds, cashew, pistachio, walnuts, hazelnuts ...) - no salted bran nuts	○	○	○	○	○
Chocolate	○	○	○	○	○
Candy and other sweets	○	○	○	○	○
Chips, salty biscuits and crackers	○	○	○	○	○

Do you believe that taking probiotics (= live bacteria that, by definition, are beneficial to your health when taken in sufficient amounts) can be beneficial for your health?

- ○ Yes
- ○ No
- ○ I don't know

Have you ever taken probiotics (orally, vaginally or in any other form)?

- ○ Yes
- ○ No

If yes, how did you come across probiotics?

- ○ Recommended by a doctor
- ○ Recommended in pharmacies
- ○ Online/media (TV, radio) information
- ○ Friends, family, other
- ○ Other: ....

What did you use probiotics for? (multiple answers possible)

- ○ Immune system
- ○ Digest
- ○ Vaginal health
- ○ Stress/mental health
- ○ Allergy
- ○ Skin
- ○ Respiratory diseases
- ○ Other: ....

Which probiotic products did you use? Please also give the name of the product

....

General health

Has your overall health status changed?
- Yes, please specify:................
- No

Have you had any antibiotic or antifungal treatment in the past month (also report vaginal antibiotics and antifungal such as e.g. gynodaktarin)?
- Yes, which one?.................
- No

Have you taken any new medication in the last month?
- Yes, which one?...............
- No

Did you feel gloomier and/or more stressed than usual on average over the past month?
- Yes
- Same
- Less than previously indicated

Reproductive and sexual health

At what age did your monthly bleeding start?
- Age in years: ....
- I don't know

Did you have sex in the past week? By this we mean all kinds of stimulation of the vagina, vulva, clitoris and anus, including manual, oral, vaginal and anal sex, masturbation,...(with or without vaginal penetration)
- Yes
- No

If yes, have you received or performed the following sexual acts on yourself (masturbation) in the past 24h?
- Manual sex
- Oral sex
- Anal sex
- Vaginal sex
- Without penetration
- Penetration with penis
- Penetration with finger(s)/hand
- Penetration with toys
- Penetration with other: ...
- Other: ....

Are you currently breastfeeding?
- Yes
- No

What drug have you used in the last month to avoid getting pregnant? Please also indicate which brand you used (multiple answers possible)

- Contraceptive pill:........................
- Mini pill:........................
- Lancing pill:........................
- Contraceptive patch (e.g. Evra) :........................
- Contraceptive ring (e.g. Nuvaring) :........................
- Hormonal implant (e.g. Implanon) :........................
- Hormone IUD (e.g. Mirena): ....
- Copper IUD:........................
- Condom:........................
- Pessary (= rubber dome) :........................
- Periodic abstinence (= not having sex in fertile period)
- Coitus interruptus (= withdrawing penis before ejaculation)
- Other:........................
- No

Why haven't you used a contraceptive method in the past month? (multiple answers possible)

- I wasn't having sex
- I had sex but couldn't get pregnant from it
- I am infertile or less fertile
- I would like to get pregnant
- Contraception is expensive
- My partner is infertile or less fertile
- My partner uses contraception
- My partner has been sterilised
- I have been sterilised
- I don't like using
- My partner does not want to use contraception
- The use of contraception is not the norm within my culture
- I do not use contraception for religious reasons
- I have a female partner
- Other:...................

Have you had a period in the last 6 months?

- Yes
- No
- I don't know

If yes, when was your first day of your last period?

- The first day of my last period was (dd/mm/yyyy): ..../........./.........
- I don't know

Have you used the following products in the last 48h?

- Tampon

- Monthly pads
- Menstrual cup
- Panty liners
- Other: ....
- No

Have you suffered from one or more complaints of your vagina or urogenital system in the last week (multiple answers possible)?

- No
- Redness
- Swelling
- Pain (general)
- Itch
- Burning sensation
- Increase in vaginal discharge (other than normal monthly discharge)
- Change in vaginal discharge (different colour, unpleasant smell)
- Pain during sexual contact
- Cystitis
- Other:.................................

Which of the following products have you used in the past 48h? (multiple answers possible)

- Vaginal soap (e.g. Lactacyd femina, Multi-Gyn, Sebamed, ...)
- Vaginal probiotics (e.g. Physioflor, Pro-Ven, Mucogyne, Gynoflor, ...)
- Vaginal shower
- None of the above

Personal hygiene

Did you in the last 24h	YES	NO
Take a bath	○	○
Take a shower	○	○
Only washed your vaginal area at the sink, bidet, Japanese toilet, etc.?	○	○
Use wet wipes	○	○
Sleep with underwear	○	○

How to clean your vagina after going to the toilet ?

- Wiping from front to back
- Wiping from back to front
- I do both
- Without wiping
- Other:...........

After visiting the toilet, I use:

- Mostly toilet paper
- Usually a damp cloth

- Mostly water and my hands
- Mostly a flannel
- Other: ....

Which underwear have you worn in the past week (Multiple answers possible)?
- Satin
- Cotton
- Synthetic, Lycra
- Synthetic, Polyamide
- Synthetic, Polyester
- Synthetic, I don't know
- Bamboo
- Other:............
- Don't know

How regularly do you change your underwear?
- Several times a day
- Every day
- Several times a week
- Every week
- Less than once a week
- I don't wear underwear

Did you go swimming in the 24 h before taking your vaginal swab?(Multiple answers possible)
- Yes, private pool
- Yes, Public swimming pool
- Yes, Sea
- Yes, Swimming pond
- Yes, River
- Yes, Jacuzzi
- Yes, Other: ....
- No

From your own experience, do you have anything you want to share that you think is important for your vaginal health? Your tips & tricks for getting rid of a recurring vaginal infection? Topics you think are important that we have not yet addressed/questioned?

....

....

Do you have any other comments for us after completing this survey?

....

....

<u>Confidentiality</u>

The protection of personal data is determined by law by the Privacy Commission. All information is kept confidential in accordance with the Privacy Act. Participants will be identified by code. If the results of this study are published in a report or scientific journal, no name will be mentioned.
