# Supplementary material for: A citizen-science-enabled catalogue of the vaginal microbiome and associated factors
Source: Nat Microbiol. 2023 Oct 26;8(11):2183–95. doi: 10.1038/s41564-023-01500-0 (PMC10627828; doi:10.1038/s41564-023-01500-0)
Supplement: Supplementary file 2 — Reporting summary [file 41564_2023_1500_MOESM2_ESM.pdf]

nature portfolio

Corresponding author(s): Sarah Lebeer

Last updated by author(s): Aug 29, 2023

Reporting Summary

Nature Portfolio wishes to improve the reproducibility of the work that we publish. This form provides structure for consistency and transparency in reporting. For further information on Nature Portfolio policies, see our Editorial Policies and the Editorial Policy Checklist.

Statistics

For all statistical analyses, confirm that the following items are present in the figure legend, table legend, main text, or Methods section.

n/a	Confirmed	
☐	☒	The exact sample size (*n*) for each experimental group/condition, given as a discrete number and unit of measurement
☐	☒	A statement on whether measurements were taken from distinct samples or whether the same sample was measured repeatedly
☐	☒	The statistical test(s) used AND whether they are one- or two-sided<br>*Only common tests should be described solely by name; describe more complex techniques in the Methods section.*
☐	☒	A description of all covariates tested
☐	☒	A description of any assumptions or corrections, such as tests of normality and adjustment for multiple comparisons
☐	☒	A full description of the statistical parameters including central tendency (e.g. means) or other basic estimates (e.g. regression coefficient) AND variation (e.g. standard deviation) or associated estimates of uncertainty (e.g. confidence intervals)
☐	☒	For null hypothesis testing, the test statistic (e.g. *F*, *t*, *r*) with confidence intervals, effect sizes, degrees of freedom and *P* value noted<br>*Give P values as exact values whenever suitable.*
☒	☐	For Bayesian analysis, information on the choice of priors and Markov chain Monte Carlo settings
☒	☐	For hierarchical and complex designs, identification of the appropriate level for tests and full reporting of outcomes
☐	☒	Estimates of effect sizes (e.g. Cohen's *d*, Pearson's *r*), indicating how they were calculated

Our web collection on statistics for biologists contains articles on many of the points above.

Software and code

Policy information about availability of computer code

Data collection	Short-read sequencing (amplicon) data was processed using an in-house script that uses the open-source R package DADA2, v1.6.0. Script available at the following DOI: https://doi.org/10.6084/m9.figshare.23184332
Data analysis	The read count table was processed with custom scripts using R v4.1.1, the tidyverse v1.3.0, and the in-house package tidyamplicons, v0.2.1. Statistical tests were performed using custom python scripts.<br>In-house toolkits are provided at https://github.com/thiesgehrmann/multidiffabundance & https:// https://github.com/Swittouck/tidyamplicons<br>All pipeline and analysis code is provided on github and at the following DOI: https://doi.org/10.6084/m9.figshare.23184332The in-house amplicon sequencing toolkit used is available at https://github.com/SWittouck/tidyamplicons. The tool to perform differential abundance with multiple different testing tools is available at https://github.com/thiesgehrmann/multidiffabundance. All other scripts used for data processing and analysis are hosted on GitHub and accessible at https://github.com/LebeerLab/Citizen-science-map-of-the-vaginal-microbiome.

For manuscripts utilizing custom algorithms or software that are central to the research but not yet described in published literature, software must be made available to editors and reviewers. We strongly encourage code deposition in a community repository (e.g. GitHub). See the Nature Portfolio guidelines for submitting code & software for further information.

Data

Policy information about availability of data

All manuscripts must include a data availability statement. This statement should provide the following information, where applicable:

- Accession codes, unique identifiers, or web links for publicly available datasets
- A description of any restrictions on data availability
- For clinical datasets or third party data, please ensure that the statement adheres to our policy

Sequencing data are available at the European Nucleotide Archive (ENA) under bioproject PRJEB50407. Metadata are available via the access-controlled European Genome-Phenome Archive (EGA) hosted by EMBL-EBI at https://web2.ega-archive.org/datasets/EGAD00001009890.

Human research participants

Policy information about studies involving human research participants and Sex and Gender in Research.

Reporting on sex and gender	The study was directed towards women, or more specific persons with a vagina. The only inclusions criteria were having a vagina, not being pregnant and at least 18 years old.
Population characteristics	The mean age and body mass indexes (BMIs) of the included participants were 31.8 ± 9.5 years and 24.3 ± 4.6 kg/m2, respectively (Figure 1d).<br>The call was directed towards the general female population outside a clinical setting. Indeed, 70% % (2278/3254) of the women did not report a single vaginal health complaint at the time of sampling based on the questionnaire data. 18.3% (594/3254) had one self-reported vaginal complaint, ranging from redness, dryness, odor, increased and/or discoloration of discharge, pain during intercourse, itching, swelling, burning, to urinary infection. Only 6.8% (222/3254) and 2.6% (85/3254) reported two, or three complaint respectively. Nevertheless, 54% (2537 /4682 4282) and 72% (3396/4682) of the participants answered to have at least once experienced a fungal infection or bladder infection.
Recruitment	The study was approved by the Ethical Committee of the Antwerp University Hospital/University of Antwerp (B300201942076) and registered online at clinicaltrials.gov with the unique identifier NCT04319536. The call for participants was launched on March 24th, 2020 and participants were recruited through calls on social media (facebook, twitter, instagram) with the help of influencers who support the project, and via an announcement in the regular media (newspapers, magazines and radio). Within ten days, 6,007 women registered through the Isala website (https://isala.be/en/) by answering five questions on age, postal code, previous pregnancies, residence country in their first three years and contraceptive use. After obtaining a digital informed consent, these participants were invited to fill out a large online survey that included relevant and GDPR-compliant questions on the Qualtrics platform (Qualtrics, Provo, UT, USA). (suppl info). The 4,682 participants that filled out the entire survey were invited to fill out their address on the website to receive an Isala self-sampling kit. Eventually, 4,106 self-sampling kits were sent out and 81.5% of the kits were returned to the University of Antwerp between July-October 2020. Two vaginal swabs were self-collected in a standardized way by non-pregnant participants (n = 3,323) and 3,294 participants filled out a short follow-up survey with 39 questions within 24 hours of sampling.
Ethics oversight	Ethical Committee of the Antwerp University Hospital/University of Antwerp (B300201942076)

Note that full information on the approval of the study protocol must also be provided in the manuscript.

Field-specific reporting

Please select the one below that is the best fit for your research. If you are not sure, read the appropriate sections before making your selection.

☒ Life sciences ☐ Behavioural & social sciences ☐ Ecological, evolutionary & environmental sciences

For a reference copy of the document with all sections, see nature.com/documents/nr-reporting-summary-flat.pdf

Life sciences study design

All studies must disclose on these points even when the disclosure is negative.

Sample size	Sample size was chosen based on the number of people that reacted to the initial call for participants, and based on the budget available for microbial community DNA sequencing.
Data exclusions	Samples from participants that were pregnant at time of sampling were removed, as well as samples with a low DNA concentration and samples with fewer than 2,000 reads. Only samples with complete survey answers where included in analyses.
Replication	The TSNE analysis of continuity of CSTs and taxon-taxon correlation analysis was also performed on the public VALENCIA and VaHMP datasets and the main trends were reproduced. We also inspected the 264 vaginal metagenomes of the VIRGO metastudy for the presence of

	Limosilactobacillus species. Finally, our amplicon sequencing pipeline was validated by sequencing a subset of 18 samples with shotgun sequencing; the results were very similar.
Randomization	There was no randomization since this was an observational (not an experimental) study. All association tests were controlled for the covariate age and for technical covariates such as the sequencing run.
Blinding	Since this was an observational study, blinding was not applicable.

Reporting for specific materials, systems and methods

We require information from authors about some types of materials, experimental systems and methods used in many studies. Here, indicate whether each material, system or method listed is relevant to your study. If you are not sure if a list item applies to your research, read the appropriate section before selecting a response.

Materials & experimental systems

n/a	Involved in the study
☒	☐ Antibodies
☒	☐ Eukaryotic cell lines
☒	☐ Palaeontology and archaeology
☒	☐ Animals and other organisms
☒	☐ Clinical data
☒	☐ Dual use research of concern

Methods

n/a	Involved in the study
☒	☐ ChIP-seq
☒	☐ Flow cytometry
☒	☐ MRI-based neuroimaging
